# Validation of key Arctic energy and water budget components in CMIP6

**DOI:** 10.1007/s00382-024-07105-5

**Published:** 2024-02-03

**Authors:** Susanna Winkelbauer, Michael Mayer, Leopold Haimberger

**Affiliations:** 1 Department of Meteorology and Geophysics, University of Vienna, Vienna, Austria; 2 b.geos, Korneuburg, Austria; 3 European Centre for Medium-Range Weather Forecasts, Bonn, Germany; 4 Austrian Polar Research Institute (APRI), Vienna, Austria

**Keywords:** Arctic, Energy budget, Water budget, CMIP6, Validation, Bias

## Abstract

We investigate historical simulations of relevant components of the Arctic energy and water budgets for 39 Coupled Model Intercomparison Project Phase 6 (CMIP6) models and validate them against observation-based estimates. We look at simulated seasonal cycles, long-term averages and trends of lateral transports and storage rates in atmosphere and ocean as well as vertical fluxes at top-of-atmosphere and the surface. We find large inter-model spreads and systematic biases in the representation of annual cycles and long-term averages. Surface freshwater fluxes associated with precipitation and evaporation as well as runoff from Arctic lands tend to be overestimated by most CMIP6 models and about two thirds of the analysed models feature an early timing bias of one month in the runoff cycle phase, related to an early snow melt bias and the lack of realistic river routing schemes. Further, large biases are found for oceanic volume transports, partly because data required for accurate oceanic transport computations has not been archived. Biases are also present in the simulated energy budget components. The net vertical energy flux out of the ocean at the Arctic surface as well as poleward oceanic heat transports are systematically underestimated by all models. We find strong anti-correlation between average oceanic heat transports and mean sea ice cover, atmospheric heat transports, and also the long-term ocean warming rate. The latter strongly suggests that accurate depiction of the mean state is a prerequisite for realistic projections of future warming of the Arctic. Our diagnostics also provide useful process-based metrics for model selection to constrain projections.

**Supplementary Information:**

The online version contains supplementary material available at 10.1007/s00382-024-07105-5.

## Introduction

The Arctic has undergone major changes in recent decades due to climate warming, with important implications not only for the Arctic itself, but also for the global climate system. Various feedback mechanisms like the ice-albedo feedback or the Planck feedback (Goosse et al. 2018) lead to a faster warming of the Polar regions compared to the globe. While earlier studies report a warming about twice the global average (Serreze et al. 2009; Walsh 2014), more recent observational datasets suggest an even stronger warming about 4 times the global average (Rantanen et al. 2022). Rising temperatures provoke the degradation of permafrost (Rowland et al. 2010), thawing of the Greenland ice sheet (Mouginot et al. 2019) and sea ice melt (Stroeve and Notz 2018). The decline in sea ice area and thickness has been particularly prominent in recent decades (Kwok 2018) and is caused by both atmospheric and oceanic processes (Docquier and Koenigk 2021). Northward heat transports in both the atmosphere and ocean counterbalance an average net loss of energy to space in the Arctic. Variability and trends in those transports have major impacts on the state and change of the Arctic system, including sea ice, the atmosphere and the ocean (Docquier and Koenigk 2021).

Arctic warming also has a strong impact on the Arctic water balance, leading to an increase of runoff from land areas and the Greenland ice sheet as well as increases in precipitation. The reasons for enhanced Arctic precipitation changes are still under debate. While earlier studies attribute increases in area-integrated evaporation due to increased open water areas together with enhanced moisture transports from lower latitudes (Bintanja and Selten 2014), more recent studies argue that the changes are consequences of the Planck feedback and therefore energetically driven (Pithan and Jung 2021; Bonan et al. 2023)

The effects of Arctic warming are not only limited to the Arctic—the melting of glaciers and the Greenland ice sheet contribute to rapid sea-level rise around the globe (e.g., Moon et al. 2018; Box et al. 2022) and the release of larger amounts of freshwater to the Atlantic Ocean (Haine et al. 2015) could have major implications for the oceanic circulation at a global scale. Further, there is contrasting evidence regarding the hypothesis that a reduction in the meridional temperature gradient might affect weather and climate in the mid-latitudes (e.g., Blackport and Screen 2020; Coumou et al. 2018; Francis and Vavrus 2012; Screen and Simmonds 2013)

Thus, the Arctic represents a complex system marked by tight couplings between atmosphere, ocean and sea ice, encompassing processes on various spatial and temporal scales. Analyzing the Arctic energy and water budgets is crucial to understand the physical processes of the system as well as the couplings between its components and to comprehend the pronounced warming trend and the resulting impacts on the Arctic system itself and globally. Further, improved process understanding and accurate validation data is needed to develop and enhance climate models and subsequently improve our knowledge of future Arctic change.

The development of the Coupled Model Intercomparison Project, a global collaborative initiative with its latest generation CMIP6 (Eyring et al. 2016), whose data are used to i.a. underpin the 6th Assessment Report of the Intergovernmental Panel on Climate Change (e.g., Fox-Kemper et al. 2021), helps to assess projected future changes under various greenhouse gas emission scenarios and is essential in understanding and quantifying the strength and the effects of climate change. However, the complex interactions between atmosphere, ocean and sea ice pose a major challenge to Arctic climate simulations and introduce large uncertainties and biases (Cai et al. 2021; Knutti 2008). This raises the need for a thorough evaluation of historical climate model simulations against observations in order to detect model biases, find potential shortcomings and improve our confidence in future projections.

However, due to the harsh environmental conditions and sheer remoteness, measurements in the polar regions are relatively sparse (Khosravi et al. 2022), complicating especially ocean and sea ice diagnostics. Satellite observations help in the quantification of surface properties, however in-situ data to assess subsurface properties, like vertically resolved temperatures in the ocean, are limited.

Over the past years, the usage of ocean reanalyses (ORAs) proved to be useful to study past ocean states, long-term climate trends and investigate ocean variability (Storto et al. 2019b; von Schuckmann et al. 2020; Mayer et al. 2021c, 2022). However, as their reliability depends i.a. on the quality and quantity of observational data assimilated into the models, the reanalyses are affected by data paucity in the Arctic. Nevertheless, Mayer et al. (2021c) show that ORAs realistically represents observed trends and temporal variabilities of ocean heat content (OHC) in the Norwegian Sea. Cheng et al. (2022) find that the uncertainty of Arctic OHC is larger than for the other world basins, however they still find consistent trends for Arctic OHC between observations and a reanalysis product. Mayer et al. (2022) find a good agreement between ORAs and observations of the variability of ocean heat transport (OHT) anomalies into the Arctic Mediterranean, but they find OHT to be biased small by about 14%. In general, OHC is more strongly constrained in ORAs than oceanic transports and hence are deemed to be more reliable. A largely observation-based estimate of OHTs is provided by Tsubouchi et al. (2018), who derive transport estimates from moorings in a mass-consistent way, creating a largely model-independent estimate of Arctic OHTs.

Serreze et al. (2009) provide holistic estimates of annual cycles and long-term means of the coupled Arctic energy and water budget. However, their results contained inconsistencies of the various terms as indicated by large budget residuals, which is likely related to inaccurate data and suboptimal diagnostic methods (such as a biased atmospheric budget framework, see Mayer et al. 2017). Therefore, Mayer et al. (2019) combine transports from Tsubouchi et al. (2018) with state-of-the-art reanalyses and other observational products and provide updated and improved, consistent estimates of the coupled Arctic energy budget for the period 2005–2009. Similarly Winkelbauer et al. (2022) provide observationally constrained estimates of the key components of the Arctic water budget using observational datasets as well as reanalyses for 1993–2019.

In this study, we will use the observationally constrained estimates from Mayer et al. (2019) and Winkelbauer et al. (2022) as well as updated estimates from observations and reanalyses to evaluate a large ensemble of CMIP6 models. We aim to analyse the models’ ability to accurately simulate some of the key components of the Arctic energy and water budgets and analyse the simulated long-term averages and seasonal cycles of the various energy and water cycle variables and their connections to understand typical model biases.

The paper is structured as follows. Section 2 introduces the main energy and water budget equations and describes the numerical methods used for calculating them, and Sect. 3 describes the data sets analysed and the study area. The results are presented in Sect. 4 and are divided into water (Sect. 4.1) and energy (Sect. 4.2) budget analyses. Conclusions and discussions follow in Sect. 5.

## Methods

In this section we formulate the vertically integrated energy and water balance equations for the Arctic and describe the analytical methods used.

### Energy and water budgets

For the Arctic energy cycle, we follow Mayer et al. (2019) and define the equation for the total energy budget of the atmosphere as

1 $$\begin{aligned} F_{S}=F_{TOA}-AET-\nabla \cdot F_A-L_{f}(T_{P})P_{snow} \end{aligned}$$

with the net (turbulent plus net radiative) vertical energy flux at the surface F$_{S}$, the net radiation at the top of the atmosphere F$_{TOA}$, the atmospheric total energy tendency AET and the divergence of vertically integrated lateral atmospheric energy transports $\nabla \cdot F_A$, which is equal to atmospheric energy transports over the lateral boundaries (AHT). The last term represents the cooling of the surface due to falling snow and consists of the latent heat of fusion L$_f$ (− 0.3337 $\times$ 10$^6$ J $\hbox {kg}^{-1}$ ) and the snowfall rate P$_{snow}$. Vertical fluxes are defined as positive downwards. The energy budget equation for an ocean-sea ice column reads as follows:

2 $$\begin{aligned} F_{S}= & {} OHCT+\nabla \cdot F_O + MET + IHCT + \nabla \cdot F_I\nonumber \\{} & {} - L_f(T_p)P_{snow} + L_f\rho _{snow}\frac{\partial d_{snow}}{\partial t} \end{aligned}$$

with the temporal tendency of ocean heat content OHCT, the divergence of vertically integrated ocean heat transport $\nabla \cdot F_O$, the sea ice melt energy tendency MET (i.e. the energy absorbed or released during melt and freeze, respectively, computed as the product of monthly sea ice thickness change and L$_f$), the sea ice sensible heat content tendency IHCT, the divergence of latent heat transport associated with sea ice transports $\nabla \cdot F_I$ and the snowfall term. The last term describes latent heat changes in conjunction with changes in grid-point-averaged snow thickness (d$_{snow}$).

For the oceanic water budget equation we follow Winkelbauer et al. (2022) and formulate it in its volumetric form:

3 $$\begin{aligned} \Delta S_O = P+ET+R-\nabla \cdot F_{vol} \end{aligned}$$

with the change of ocean volume denoted as $\Delta S_O$, the surface water fluxes precipitation P and evapotranspiration ET (counted positive downward), runoff from surrounding land areas R and the divergence of lateral oceanic volume fluxes $\nabla \cdot F_{vol}$.

Furthermore, following Gauss’s divergence theorem the divergence terms in equations 2 and 3 can be replaced by transports of energy and volume across the lateral boundaries when considering closed oceanic regions.

### Oceanic transports

Oceanic transports of volume (OVT), heat (OHT) and ice (OIT) through a given strait are defined as follows:

4 $$\begin{aligned} OVT= & {} \int _{x_s}^{x_e}\int _{0}^{z(x)} \vec {v}_o(x,z)\cdot \vec {n}\,dz\,dx \end{aligned}$$

5 $$\begin{aligned} OIT= & {} \int _{x_s}^{x_e} d(x)\vec {v}_i(x)\cdot \vec {n}\,dx \end{aligned}$$

6 $$\begin{aligned} OHT= & {} c_p \rho \int _{x_s}^{x_e}\int _{0}^{z(x)} (\theta (x,z)-\theta _{ref}) \vec {v}_o(x,z)\cdot \vec {n}\,dz\,dx \end{aligned}$$

where $\vec {v}_o$ is the velocity vector of the oceanic flow and $\vec {n}$ is the vector normal to the strait. Furthermore, x defines the width along the strait, with the straits’ starting point x$_s$ and the end point x$_e$. The straits’ depth is given by z, where x and z together form the cross sectional area of the strait. Ice transports are calculated by integrating the cross-sectional ice velocity $\vec {v}_i$ over the grid point average ice depth (d) and integrating over the section. Latent heat transports into the study area through ice exports (IHT) are then estimated by multiplying OIT with the sea ice density (assumed constant at 928 $\hbox {kgm}^{-3}$) and the latent heat of fusion L$_f$ (− 0.3337 $\times$ 10$^6$ J $\hbox {kg}^{-1}$). Computation of heat transports requires potential temperature $\theta$, the specific heat of seawater $c_p$ and the density of seawater $\rho$. Throughout this study, $c_p$ and $\rho$ are kept constant at 3996 $\hbox {Jkg}^{-1}$ $\hbox {K}^{-1}$ and 1026 $\hbox {kgm}^{-3}$, respectively, because variations in $c_p$ and $\rho$ tend to compensate each other and together lead to only small changes in the computed heat transports (Fasullo and Trenberth 2008) which are neglected in the context of this study.

As discussed by Schauer and Beszczynska-Möller (2009), unambiguous heat transports would actually demand closed volume transports through the examined straits, which is not the case for the single straits considered here, and only approximately satisfied for the total oceanic transport through all straits. As a result, heat transports have to be calculated relative to a reference temperature $\theta _{ref}$, which should represent the mean temperature of the assessed flow. Strictly speaking this reference temperature should vary spatially and temporally according to the investigated flow (Bacon et al. 2015). While changes in the reference temperature have only minor effects on the net Arctic transports (not shown), they are larger for transports through individual straits and may become significant the stronger $\theta _{ref}$ changes. However, to simplify the analysis we follow e.g. Tsubouchi et al. (2012), Tsubouchi et al. (2018), Muilwijk et al. (2018), Shu et al. (2022), Heuzé et al. (2023) and calculate all heat transports relative to a 0$^\circ C$ reference. Usage of the same reference temperature for all models and straits also allows for better inter-comparisons with one another (Muilwijk et al. 2018).

Transports must be calculated on the native grids of the models to maintain the conservation properties of the models. However, ocean models often use curvilinear grids where the North Pole is placed over land areas to avoid singularities over the ocean. The number of poles (tri- vs. dipolar), the exact location of the poles, and the Arakawa partition vary between models, resulting in a large number of different grid types, making it difficult to compare models and with observations. We have developed two methods for calculating accurate ocean transports on different CMIP6 model grids, which are described in Winkelbauer et al. (2023) and are available via the Python package StraitFlux (Winkelbauer 2023).

**Fig. 1:**
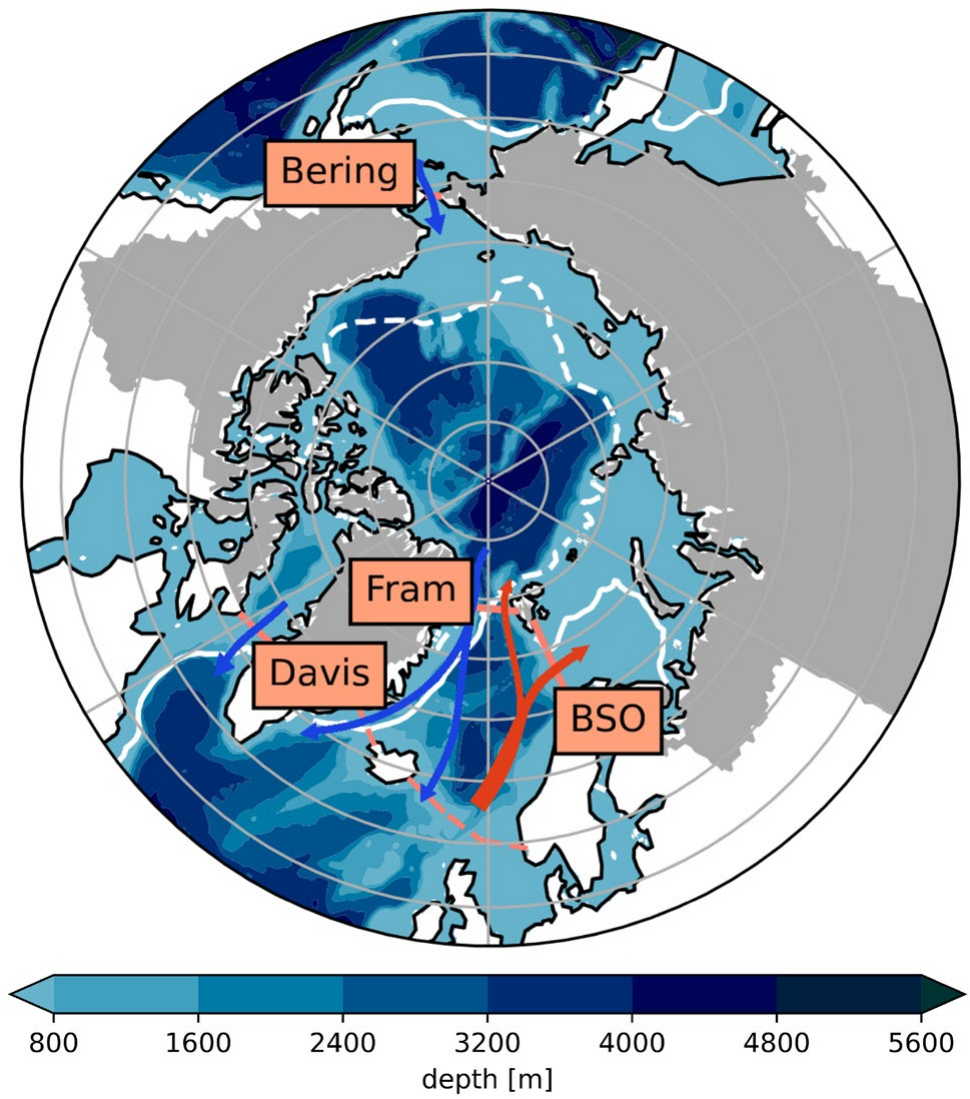
Map of the main study area, consisting of the oceanic area bounded by the main Arctic gateways (indicated by solid orange lines; corresponds to $11.3\times 10^6$ $\hbox {km}^2$) and the terrestrial drainage area (grey shading; corresponds to $18.2 \times 10^6$ $\hbox {km}^2$ for mainlands and islands and additional $0.95 \times 10^6$ $\hbox {km}^2$ for Greenland). The orange dashed line indicates the position of the Greenland-Scotland Ridge, which bounds, together with Fram Strait and BSO, the region of the Nordic Seas. Additionally, the main currents flowing in and out of the Arctic (red and blue arrows for warm inflow and cold outflow, respectively) and 1993–2014 mean March (white solid) and September (white dashed) 30% sea ice concentration lines (taken from the GREP reanalyses ensemble) are shown. Shading in the oceanic areas indicates the bathymetry

Net Arctic transports are calculated as the sum of transports through Fram Strait, Davis Strait, the Barents Sea Opening and Bering Strait (see Fig. 1 below for the location of the cross-sections).

### Metrics

To validate CMIP6 output against observations, scalar quantities are regridded to regular grids in the resolution of the available observation-based data (0.25$^{\circ }$ ✕ 0.25$^{\circ }$ and 1$^{\circ }$ ✕ 1$^{\circ }$ grids). However, quantities using vector-based components are computed on the respective native grids of the models to avoid any errors associated with the interpolation of vector quantities. Spatial averages are calculated over the Arctic areas as defined in Fig. 1 and long-term average seasonal cycles are determined over the 1993–2014 period.

We calculate decadal trends by applying a linear regression to the monthly anomaly (i.e., deseasonalized) time series. Significance is determined by the Wald test with a t-distribution, with p-values less than 0.05 considered significant. Inter-model correlations are calculated using Pearson’s correlation coefficient r and to assess seasonal model performance we use normalised mean errors (nME). The normalisation for each variable is done using the largest error of all models for the variable in question to facilitate inter-model comparisons. For instance, the nME for model j over N years (whereby annual averages are calculated using only the assessed season, e.g. DJF for winter) is calculated as follows:

7 $$\begin{aligned} nME_j=\frac{\sum _{i=1}^{N}(data_{j,i} - reference_{j,i})}{MAX_k^K(nME_k)} \end{aligned}$$

To determine sampling errors of long-term averages that can arise, e.g., from different states of natural variability modes in the model runs compared to observations, we use a bootstraping approach of random sampling with replacement. Thus, for every model and variable we calculate 1000 long-term averages of the desired period (e.g. 22 years for the 1993–2014 period) out of randomly drawn annual averages within the most recent decades (1980–2014). The sampling error is then estimated as 2-sigma standard deviation from the distribution of the randomly sampled long-term averages.

Confidence ellipses for two-dimensional datasets (see all scatter-diagrams in Sec. 4 and the supplementary material) are calculated using the Pearson correlation coefficient as described at https://carstenschelp.github.io/2018/09/14/Plot_Confidence_Ellipse_001.html. They are determined for the 2-sigma standard deviation and therefore encompass about 95% of all values in the 2D space.

## Data and study domain

### CMIP6 models

We use monthly output from 39 models that participated in the Climate Model Intercomparison Project Phase 6 [CMIP6, Eyring et al. (2016)]. Table 1 lists all the models used in this study, including their modelling components, and provides links to key references. We use historical model runs for 37 models and the hist-1950 model run for EC-Earth3P-HR and HadGEM3-GC31-MM. We use one ensemble member per model and choose the first available member per model, r1i1p1f2 for CNRM-CM6-1, CNRM-CM6-1-HR, MIROC-ES2L and UKESM1-0-LL, r1i1p1f3 for HadGEM3-GC31-LL and HadGEM3-GC31-MM, r1i1p2f1 for EC-Earth3P-HR and r1i1p1f1 for the remaining 32 models. The models have different horizontal and vertical resolutions (please refer to the individual model documentation listed in Tab. 1) and differ in their modelling components for atmosphere, land, ocean and sea ice. However, the models are not completely independent and often overlap in one or more modelling components. Therefore, when calculating the multi-model mean (MMM), models should ideally be preselected to avoid overlapping components or weighted with respect to their independence and performance (Brunner et al. 2020). Hence, results might differ when compiling a model ensemble that maximizes independence of its members, but this is not the focus of this study.

All data are obtained from the Earth System Grid Federation (ESGF) website (https://esgf-node.llnl.gov/search/cmip6/). We assess different components of the energy and water budgets. Table 2 lists the variables used in this study, not all variables are available for all models, therefore the number of available models (n) and a list of missing models (numbers correspond to indices in Table 1) are also given in Table 2. The variables listed in Table 2 are used to derive the main budget components represented by Eqs. 1 to 3, such as F$_s$, F$_{TOA}$, AET, OHCT, MET, OHT, OVT and OIT. F$_{TOA}$ and F$_s$ are calculated directly using all available radiative and turbulent heat flux components (see Table 2) and oceanic transports are calculated using StraitFlux (Winkelbauer et al. 2023). Heat content tendencies in the ocean (OHCT) and sea ice (MET) are calculated from sea water potential temperature and sea ice thicknesse respectively, using a Theil-Sen trend estimator. Atmospheric energy tendencies (AET) are calculated on temperature and humidity levels using central differences of monthly mean values and the atmospheric heat transport AHT, which is equal to the divergence term $\nabla \cdot F_A$, is estimated indirectly using equation 1.

Sea ice extent was calculated similarly to Shu et al. (2020) as the area of all grid cells with sea ice concentration (*siconc*) greater than 15%. For sea ice thickness we either use the variable *sivol* or multiply *sithick* with *siconc*, depending on the availability of the variable through ESGF.

**Table 1:** List of models included in the analysis, their modelling components and links to relevant references

i	Model name	Atmosphere	Land	Ocean	Ice	References
1	ACCESS-CM2	UM10.6 GA7.1	CABLE2.5	MOM5.1	CICE5.1.2	Bi et al. (2020)
2	ACCESS-ESM1-5	UM7.3 GA1	CABLE2.4	MOM5.1	CICE4.1	Ziehn et al. (2020)
3	BCC-CSM2-MR	BCC-AGCM3-MR	BCC AVIM2	MOM4	SIS2	Wu et al. (2019)
4	BCC-ESM1	BCC-AGCM3-Chem	BCC AVIM2	MOM4	SIS2	Wu et al. (2019)
5	CAMS-CSM1-0	ECHAM5-CAMS	CoLM 1.0	MOM4	SIS1	Chen et al. (2019)
6	CanESM5	CanAM5	CLASS3.6-CTEM	NEMO3.4	LIM2	Swart et al. (2019)
7	CAS-ESM2-0	IAP AGCM5.0	CoLM	LICOM2.0	CICE4.0E	Zhang et al. (2020)
8	CESM2	CAM6	CLM5	POP2	CICE5.1	Danabasoglu et al. (2020)
9	CESM2-WACCM	WACCM6	CLM5	POP2	CICE5.1	Danabasoglu et al. (2020)
10	CMCC-CM2-HR4	CAM4	CLM4.5	NEMO3.6	CICE4.0	N/A
11	CMCC-CM2-SR5	CAM5.3	CLM4.5	NEMO3.6	CICE4.0	N/A
12	CNRM-CM6-1	ARPEGE-Clim6.3	Surfex 8.0c	NEMO3.6	Gelato 6.1	Voldoire et al. (2019)
13	CNRM-CM6-1-HR	ARPEGE-Clim6.3	Surfex 8.0c	NEMO3.6	Gelato 6.1	Voldoire et al. (2019)
14	EC-Earth3P-HR	IFS cy36r4	HTESSEL	NEMO3.6	LIM3	Haarsma et al. (2020)
15	EC-Earth3	IFS cy36r4	HTESSEL	NEMO3.6	LIM3	Döscher et al. (2022)
16	FGOALS-f3-L	FAMIL2.2	CLM4.0	LICOM3.0	CICE4.0	He et al. (2019)
17	FGOALS-g3	GAMIL3	CAS-LSM	LICOM3.0	CICE4.0	Li et al. (2020)
18	FIO-ESM2-0	CAM5	CLM4.0	POP2	CICE4	Bao et al. (2020)
19	GFDL-CM4	AM4.0	LM4.0	MOM6	SIS2	Held et al. (2019)
20	GFDL-ESM4	AM4.1	LM4.1	MOM6	SIS2	Dunne et al. (2020)
21	GISS-E2-1-G	GISS-E2.1	GISS LSM	GISS Ocean v1	GISS SI	Kelley et al. (2020)
22	HadGEM3-GC31-LL	UM-HG3-GA7.1	JULES-HG3-GL7.1	NEMO-HG3-GO6	CICE-HG3-GS18	Williams et al. (2018)
23	HadGEM3-GC31-MM	UM-HG3-GA7.1	JULES-HG3-GL7.1	NEMO-HG3-GO6	CICE-HG3-GS18	Williams et al. (2018)
24	INM-CM5-0	INM-AM5-0	INM-LND1	INM-OM5	INM-ICE1	N/A
25	IPSL-CM6A-LR	LMDZ	ORCHIDEE	NEMO-OPA	NEMO-LIM3	Boucher et al. (2020)
26	IPSL-CM6A-LR-INCA	LMDZ	ORCHIDEE	NEMO-OPA	NEMO-LIM3	Boucher et al. (2020)
27	KACE-1-0-G	UM-HG3-GA7.1	JULES-HG3-GL7.1	MOM4p1	CICE-HG3-GSI8	Lee et al. (2020)
28	KIOST-ESM	AM2	LM3.0	MOM5	SIS	Pak et al. (2021)
29	MIROC-ES2L	CCSR-NIES AGCM	MATSIRO6	COCO4.9	COCO4.9	Hajima et al. (2020)
30	MIROC6	CCSR AGCM	MATSIRO6	COCO4.9	COCO4.9	Tatebe et al. (2019)
31	MPI-ESM1-2-HR	ECHAM6.3	JSBACH3.20	MPIOM1.63	MPIOM1.63	Mauritsen et al. (2019)
32	MPI-ESM1-2-LR	ECHAM6.3	JSBACH3.21	MPIOM1.63	MPIOM1.63	Mauritsen et al. (2019)
33	MRI-ESM2-0	MRI-AGCM3.5	HAL 1.0	MRI.COMv4	MRI.COMv4	Yukimoto et al. (2019)
34	NESM3	ECHAM6.3	JSBACH	NEMO3.4	CICE4.1	Cao et al. (2018)
35	NorCPM1	CAM(OSLO4.1)	CLM4	MICOM1.1	CICE4	Bethke et al. (2021)
36	NorESM2-MM	CAM6	CLM5	BLOM	CICE5.1.2	Seland et al. (2020)
37	SAM0-UNICON	CAM5-UNICON	CLM4.0	POP2	CICE4.0	Park et al. (2019)
38	TaiESM1	TaiAM1	CLM4.0	POP2	CICE4	Wang et al. (2021)
39	UKESM1-0-LL	UM-HG3-GA7.1	JULES-ES− 1.0	NEMO-HG3-GO6	CICE-HG3-GS18	Sellar et al. (2019)

**Table 2:** List of all CMIP6 variables used through this study, including their units, number of available models n and the indices of missing models

Variable	Description	Unit	n	Missing models
rsus, rsds, rlus, rlds	Surface up-/downward, short-/longwave raditations	$\hbox {Wm}^{-2}$	39	–
rsut, rsdt, rlut	Toa outgoing short-/longwave and incident shortwave raditations	$\hbox {Wm}^{-2}$	39	–
hfss, hfls	Surface sensible and latent heat flux	$\hbox {Wm}^{-2}$	38	28
pr	Precipitation flux	kg $\hbox {m}^{-2}$ $\hbox {s}^{-1}$	39	–
evspsbl	Evaporation (incl. sublimation and transpiration)	kg $\hbox {m}^{-2}$ $\hbox {s}^{-1}$	38	28
mrro	Runoff flux	kg $\hbox {m}^{-2}$ $\hbox {s}^{-1}$	36	5, 34, 35
thetao	Sea water potential temperature	degC	38	27
uo,vo	Sea water x/y velocity	$\hbox {ms}^{-1}$	36	20,27,35
thkcello	Ocean cell thickness	m	26	7, 8, 9, 16, 17, 18, 21, 24, 27, 28, 33, 34, 35
sithick / sivol	Actual thickness of sea ice / total volume of sea ice divided by grid-cell area (=sithick*siconc)	m	35	17, 21, 24, 27
siconc	Percentage of grid cell covered by sea ice	%	36	20, 21, 27
siu, siv	Sea ice x/y velocity	$\hbox {ms}^{-1}$	20	7, 8, 9, 13, 16, 17, 18, 20, 21, 24, 27, 28, 29, 30, 33, 34, 35, 36, 38

All variables where downloaded through ESGF

### Observational data

To quantify the representation of the energy and water budget components in CMIP, we compare the modelled seasonal cycles and long-term averages with observation based estimates.

Winkelbauer et al. (2022) provide observationally constrained estimates of the key components of the Arctic water budget using in-situ and satellite observations as well as reanalyses, and enforcing budget closure with a variational approach. To avoid use of fluxes based on short-term forecasts from reanalyses, which are known to be biased (Trenberth et al. 2011), the net surface water flux (P-E) was derived from moisture flux divergence, which can be computed from analysed state quantities and thus is more strongly constrained by observations. We adapt results from Winkelbauer et al. (2022) to the 1993–2014 period and use them to validate seasonal cycles and trends of the freshwater input components R and P-E into the Arctic Ocean simulated by the CMIP6 models. As we also want to assess lateral oceanic transports through individual straits and for liquid water and sea ice separately, we additionally calculate oceanic transports directly from the Copernicus Marine Environment Monitoring Service (CMEMS) Global ocean Reanalysis Ensemble Product (GREP, Desportes et al. 2017; Storto et al. 2019a), an ensemble of four global ocean reanalyses: the CMCC Global Ocean Physical Reanalysis System (CGLORS, Storto and Masina 2016), the Forecasting Ocean Assimilation Model (FOAM, MacLachlan et al. 2015), Global Ocean Reanalysis and Simulation Version 4 (GLORYS2V4, Garric et al. 2017) and Ocean Reanalysis System 5 (ORAS5, Zuo et al. 2015). The GREP ensemble members use the NEMO ocean model and are all run at $1/4^{\circ }$ horizontal resolution with 75 vertical levels. They all use the same atmospheric forcing (ERA-Interim Dee et al. 2011), however there are differences in the data assimilation methods, used observational products, the reanalysis initial states, NEMO versions, the sea ice models, physical and numerical parameterizations, and air-sea flux formulations. For further details, we refer to the individual data documentations and Storto et al. (2019a). Additionally, we look into an improved version of FOAM (GloRanV14, hereinafter called FOAMv2). Unlike the other reanalyses, FOAMv2 uses a non-linear free surface scheme (NLFS), which introduces some differences when looking into seasonal cycles of volume transports (see Section 4.1.1). Further, we use mooring-derived transports from the so-called ArcGate project (Tsubouchi et al. 2012, 2018), which are available from October 2004 to May 2010.

For the energy budget, we compare the CMIP6 output to results from Mayer et al. (2019), who provide a consistent, closed estimate of the seasonal cycle of the Arctic energy budget for the period 2005–2009 using observations and reanalyses and also a variational optimization approach. They calculate energy budget terms from Eqs. 1 and 2 using satellite observations, various reanalyses and ocean reanalyses as well as oceanic transport derived from moorings. As here we assess longer time periods, i.a. to reduce sampling uncertainties, we additionally calculate the major budget components using observations and reanalyses directly: Net TOA fluxes are compared with the DEEP-C dataset (Liu et al. 2020; Allan et al. 2014,; publicly available at https://doi.org/10.17864/1947.271), a backward extension of the net TOA fluxes from the Clouds and the Earth’s Radiant Energy System-Energy Balanced and Filled (CERES-EBAF) satellite product in version 4.1 (Loeb et al. 2018), where fluxes prior to the CERES period have been reconstructed using satellite observations, atmospheric reanalysis and model simulations (Liu et al. 2020). F$_s$ is compared with inferred net surface energy fluxes derived from mass-consistent energy budgets using ERA5 data (Mayer et al. 2021b). The snowfall term in Eqs. 1 and 2 as well as atmospheric transports (via the *vertical integral of divergence of total energy flux*) and the atmospheric tendency term (using central differences on the *vertical integral of total energy*) are additionally calculated using data from the ERA5 reanalyses (Hersbach et al. 2020). Energy tendency components OHCT and MET, as well as latent heat transports associated with sea ice transports (IHT) are estimated using the GREP reanalysis ensemble and oceanic transports of heat are calculated using GREP and mooring-derived transports from ArcGate. Additionally, we use the merged data product from CryoSat2 and the Soil Moisture and Ocean Salinity satellites (CS2SMOS Ricker et al. 2017), which has not been assimilated in the used ocean reanalyses, to validate sea ice thickness and MET data.

The datasets used to estimate the major energy and water budget components are summed up in Table 3. The calculation of reference uncertainties depends on the used data sources: for P-E and R we use uncertainties provided by Winkelbauer et al. (2022), for oceanic transports as well as OHCT and MET we use the spread of the GREP ensemble and the remaining uncertainties are based on the standard deviations of monthly mean values.

While Mayer et al. (2019) and Winkelbauer et al. (2022) provide closed budgets and therefore consistent estimates of the budget components, the various other, independent data products for some of the budget components (as described above) are not expected to be fully consistent with each other and therefore budget closure for our observational reference estimates is not expected.

**Table 3:** List of datasets used to calculate the energy and water budget variables

Variable	Data	Time period
R	Adapted from Winkelbauer et al. (2022)	1993–2014
P-E	Adapted from Winkelbauer et al. (2022)	1993–2014
OVT	GREP	1993–2014
	ArcGate	10/2004–05/2010
OIT	GREP	1993–2014
	ArcGate	10/2004-05/2010
F$_{S}$	Mayer et al. (2021b)	1993–2014
	Mayer et al. (2019)	2005–2009
F$_{TOA}$	DEEPC	1993–2014
	Mayer et al. (2019)	2005–2009
OHCT	GREP	1993–2014
	Mayer et al. (2019)	2005–2009
MET	GREP	1993–2014
	CS2SMOS	October–March 2011–2014
	Mayer et al. (2019)	2005–2009
AET	ERA5	1993–2014
	Mayer et al. (2019)	2005–2009
AHT	ERA5	1993–2014
	Mayer et al. (2019)	2005–2009
OHT	GREP	1993–2014
	ArcGate	10/2004-05/2010

### Study area

We consider the Arctic Ocean, which is bounded by hydrographic mooring lines in Fram Strait, Bering Strait, Davis Strait and the Barents Sea Opening (BSO). There are also two small passages, Fury and Hecla Straits, which connect the Arctic Ocean to Hudson Bay through the Canadian Arctic Archipelago (CAA). However, as Tsubouchi et al. (2012) and Bacon et al. (2022) pointed out, volume fluxes through these passages are very small and are not considered in this study. Figure 1 shows the study area, which was chosen to be the same as in Mayer et al. (2019) and Winkelbauer et al. (2022).

To analyse water entering the ocean from the surrounding land areas, we additionally introduce the terrestrial domain, which consists of all land areas draining into the Arctic Ocean, including the CAA as well as islands along the Eurasian coast. We use the catchments as defined by Winkelbauer et al. (2022) and use the same area for all models. The total oceanic and terrestrial areas are $11.3\times 10^6$ $\hbox {km}^2$ and $18.2\times 10^6$ $\hbox {km}^2$ respectively, and Greenland provides an additional terrestrial catchment area of $0.95\times 10^6$ $\hbox {km}^2$.

## Results

### Water budget

This section looks at the main components of the Arctic water budget. We assess their long-term averages, trends and seasonal cycles.

**Fig. 2:**
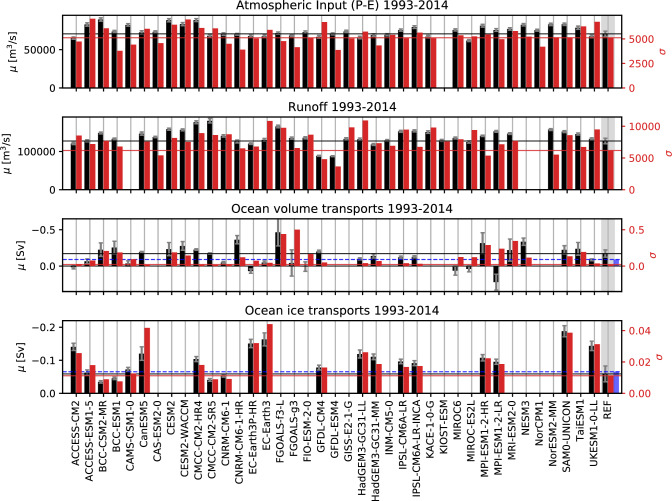
Averages (black, left axis) and standard deviations of annual averages (red, right axis) for the major Arctic water budget components. Reference values (REF) for P-E and R are taken from Winkelbauer et al. (2022). They are indicated by horizontal lines and shown on the right hand side of the panels. For the oceanic transports REF shows transports from the GREP reanalyses (1993–2014) and additionally also transports from the ArcGate project (2005–2010) are shown (blue bars and dashed lines). Error bars denote sampling errors for CMIP6 and errors calculated from the spread of used observational data for REF

Figure 2 and Table 4 show long-term averages of surface fresh water flux (P-E) and runoff (R), as well as lateral oceanic fluxes of water volume and ice for the period 1993–2014 and compared with reference values from Winkelbauer et al. (2022). Figure 2 also shows standard deviations and values in brackets in Table 4 show decadal trends. Reference values indicate a long-term mean net freshwater input to the Arctic Ocean from surface fluxes of about 208$\times$10$^3$ $\hbox {m}^3$ $\hbox {s}^{-1}$. About one-third comes from net precipitation (69.2$\times$10$^3$ $\hbox {m}^3$ $\hbox {s}^{-1}$), two-thirds from runoff from Arctic lands (127.0$\times$10$^3$ $\hbox {m}^3$ $\hbox {s}^{-1}$) and about 5% are melt water and ice discharge from the Greenlandic ice cap R$_G$ (11.9$\times$10$^3$ $\hbox {m}^3$ $\hbox {s}^{-1}$). The MMMs for oceanic P-E (38 models) and R (36 models) are about 10 % higher than our observational references. Net precipitation ranges between 63.2$\times$10$^{3}$ $\hbox {m}^3$ $\hbox {s}^{-1}$ and 91.8$\times$10$^{3}$ $\hbox {m}^3$ $\hbox {s}^{-1}$, while runoff ranges between 88.5 and 180.6$\times$10$^{3}$ $\hbox {m}^3$ $\hbox {s}^{-1}$, with the lowest values coming from GFDL-CM4 and GFDL-ESM4 and the highest values simulated by CMCC-CM2-SR5 and CMCC-CM2-HR4. Greenlandic runoff from CMIP6 models varies between 0.3 and 19.0$\times$10$^{3}$ $\hbox {m}^3$ $\hbox {s}^{-1}$ and is underestimated by most models, with the MMM being about 50% smaller than the reference value. In contrast, the CMIP6 MMM of P-E over Greenland is about 10% higher than the reference estimate and there is a clear offset between runoff and P-E for most models. As soil moisture content and the surface snow amount do not change considerably (not shown) the mass balance over Greenland does not seem to be closed for the affected models. Possible reasons may include that the catchment area used, which is assumed to be the same for all models, might omit high runoff regions, that discharge coming directly from the ice sheet and/or solid discharge is underestimated or missing or, to a lesser degree, that the models feature conservation issues over Greenland. Further analyses would be needed to get to the origin of these discrepancies, which were not in the scope of this study.

Most models agree on an increase in freshwater input to the Arctic Ocean for the period 1993–2014: 25 models show a significant increase in R (only 3 show a significant decrease) and while all models agree on increasing precipitations and evaporations, trends in precipitation prevail in most models leading to significant positive trends in oceanic P-E for 19 models (only 2 show a significant decrease). The MMMs show an increase in oceanic P-E of 2% per decade and an increase in R of 2% per decade, which is in fairly good agreement with trends in the reference data. These increases in oceanic P-E and R contribute to an increase in liquid freshwater stored in the Arctic Ocean, which has been observed (Rabe et al. 2011; Proshutinsky et al. 2009; McPhee et al. 2009) and simulated by CMIP6 models (Zanowski et al. 2021; Wang et al. 2022), and further may lead to increased oceanic freshwater exports out of the Arctic system.

Reanalyses indicate a net outflow of liquid volume from the Arctic Ocean of − 151 ± 43 mSv, while estimates derived from observation in the ArcGate project reach -91 mSv. Most CMIP6 models agree on an outflow of liquid volume out of the Arctic and the CMIP6 MMM stays within the reference estimates with − 145 mSv, however the inter-model variability is large. Some models significantly overestimate the net outflows (e.g., FGOALS-f3-L, MPI-ESM1-2-HR), while others indicate net inflows into the Arctic of up to 221 mSv (MPI-ESM1-2-LR). However, it has to be noted that diagnosed volume transports are very sensitive to the exact ocean bathymetry, where slight changes may lead to large deviations of multiple Sverdrups. As net Arctic volume transports are comparatively small values resulting from the sum of large in- and outflowing branches, small errors may lead to significant inconsistencies.

Ice volume transports have only been calculated for 20 models, with all models agreeing on an export of ice to the Atlantic. Ice transports vary between − 188 and − 35 mSv, with a MMM about 30% higher than our observational estimates (− 60 ± 19 mSv for GREP and − 65 mSv for ArcGate).

Using those precisely calculated liquid and solid transports and taking into account all volume budget terms, we are still not able to close the simulated volume budgets for the individual models. Possible reasons for those shortcomings are discussed in Sect. 4.3.

While most models simulate an increase in liquid volume exports through the Fram Strait, an increase in imports through the Barents Sea opening and a decrease in exports through the Davis Strait (not shown, see e.g. Wang et al. 2022), the trends in net volume transports for the whole Arctic vary widely between models. For ice transports, the majority of models agree on a decrease in ice exports over the considered 22-year period, with significant trends between 11 and 39% per decade and a MMM trend of 18%. Long-term averages for volume transports and trends through the individual straits are shown in Table 6.

**Table 4:** Long-term averages for the major water budget components

Units	P-E	R	R$_{G}$	OVT	OIT
	[10$^3$ $\hbox {m}^3$/s] ([frac/dec])			[mSv = 10$^3$ $\hbox {m}^3$/s] ([frac/dec])	
ACCESS-CM2	65.9 (0.04*)	122.9 (0.06*)	1.3 (0.12)	33 ($-$ 0.80)	− 141 (0.13*)
ACCESS-ESM1-5	85.1 (0.01)	130.8 (0.01*)	7.6 (0.03)	− 66 (0.03)	− 63 (0.19*)
BCC-CSM2-MR	91.8 (0.03*)	149.4 (0.01)	9.4 (0.11*)	− 226 (0.08)	− 35 (0.28*)
BCC-ESM1	75.2 (0.01)	133.8 (0.03*)	9.5 (− 0.03)	− 255 (0.33)	− 45 (0.09)
CAMS-CSM1-0	82.5 (0.00)	–	–	− 37 ($-$ 1.38*)	− 72 (0.0)
CanESM5	75.0 (0.01)	150.1 (0.03*)	8.2 (0.10)	-194 ($-$ 0.03)	− 120 (0.39*)
CAS-ESM2-0	74.7 (0.04*)	139.5 (0.00)	4.6 (0.07)	–	–
CESM2	89.8 (0.04*)	157.7 (0.02*)	4.7 (0.13)	− 234 (0.83*)	–
CESM2-WACCM	84.8 (0.03*)	159.4 ($-$ 0.00)	4.5 (0.07)	− 277 (0.48*)	–
CMCC-CM2-HR4	91.6 (0.04*)	180.6 (0.01)	3.0 (0.13)	− 227 (0.02)	-104 (0.01)
CMCC-CM2-SR5	69.3 (0.05*)	179.3 (0.01*)	4.4 (0.14)	-174 ($-$ 0.01)	− 41 (0.27*)
CNRM-CM6-1	70.9 (0.02)	145.2 (0.01)	9.7 (0.09)	-50 ($-$ 0.02)	− 53 (0.02)
CNRM-CM6-1-HR	71.2 (0.00)	129.4 (0.01*)	11.4 (0.00)	-363 (0.21)	–
EC-Earth3P-HR	68.3 (0.00)	120.0 (0.03*)	1.5 ($-$ 0.01)	70 (0.48)	− 151 (0.11*)
EC-Earth3	68.2 ($-$ 0.00)	135.7 (0.07*)	1.8 (0.12)	− 48 ($-$ 1.17)	− 163 (0.31*)
FGOALS-f3-L	72.8 (0.01)	170.0 (0.00)	2.5 (0.16)	− 465 (0.99*)	–
FGOALS-g3	70.2 ($-$ 0.00)	136.7 (0.04*)	1.5 (0.09)	− 43 (1.31*)	–
FIO-ESM2-0	73.6 (0.02)	138 (0.02*)	1.9 (0.16)	6.6 (1.05)	–
GFDL-CM4	69.4 (0.08*)	89.0 (0.03*)	0.8 (0.03)	− 200 ($-$ 0.15)	− 78 (0.18*)
GFDL-ESM4	72.2 (0.01*)	88.5 ($-$ 0.01*)	0.4 ($-$ 0.06)	–	–
GISS-E2-1-G	75.8 ($-$ 0.02*)	136.0 ($-$ 0.02*)	0.5 (0.01)	–	–
HadGEM3-GC31-LL	66.5 (0.04*)	134.6 (0.08*)	3.8 (0.27)	-99 ($-$ 0.44)	− 119 (0.31*)
HadGEM3-GC31-MM	69.5 ($-$ 0.02*)	119.8 (0.06*)	2.4 (0.18)	-139 ($-$ 0.33)	− 111 (0.16*)
INM-CM5-0	71.1 (0.00*)	131.3 (0.00)	11.9 (0.11)	–	–
IPSL-CM6A-LR	75.9 (0.01*)	156.6 (0.05*)	12.9 (0.05)	− 120 ($-$ 0.13)	− 97 (0.12*)
IPSL-CM6A-LR-INCA	80.9 (0.00)	154.4 (0.03*)	13.2 ($-$ 0.00)	− 128 ($-$ 0.09)	− 91 (0.07)
KACE-1-0-G	67.1 (0.00)	151.5 (0.01)	19.0 (0.03)	–	–
KIOST-ESM	–	132.6 (0.03*)	6.2 (0.03)	–	–
MIROC-ES2L	63.2 (0.07*)	127.5 (0.05*)	8.3 (0.04)	39 (1.76*)	–
MIROC6	76.7 (0.06*)	137.6 (0.03*)	9.9 (0.05)	64 (1.04*)	–
MPI-ESM1-2-HR	82.1 ($-$ 0.02)	141.4 (0.02*)	0.3 (0.02)	− 316 (0.47*)	− 108 (0.22*)
MPI-ESM1-2-LR	76.4 ($-$ 0.00)	153.8 ($-$ 0.01*)	0.4 (0.04)	221 ($-$ 0.53*)	− 96 (0.15*)
MRI-ESM2-0	78.0 (0.01)	149.2 (0.03*)	3.5 (0.30)	− 220 ($-$ 1.26*)	–
NESM3	84.5 (0.02*)	–		− 336 ($-$ 0.03)	–
NorCPM1	76.5 (0.04*)	–		–	–
NorESM2-MM	84.3 (0.07*)	158.8 (0.01*)	9.8 (0.05)	–	–
SAM0-UNICON	84.5 (0.04*)	154.8 (0.05*)	1.4 ($-$ 0.06)	− 224 ($-$ 0.14)	− 188 (0.15*)
TaiESM1	78.6 (− 0.00)	146.9 (0.03*)	8.0 (0.04)	− 238 (0.30*)	–
UKESM1-0-LL	68.4 (0.01)	135.5 (0.04*)	1.9 (0.37)	− 86 (− 0.33)	− 144 (0.27*)
**MMM**	75.9 (0.02*)	141.1 (0.02*)	5.6 (0.06)	− 145 (0.66)	− 101 (0.18*)
**REF**	69.2 ± 2.5 (0.02*)	127.0 ± 1.1 (0.02*)	11.9 ± 0.4 (0.13*)	− 151 ± 43 ($-$ 0.00) / − 91$^A$	− 60 (0.19*) / − 65$^A$
Winkelbauer et al. (2022)	—"—	—"—	—"—	− 207	

The MMM is calculated using all available models and REF denotes the observation based reference values. Values in brackets show decadal relative trends ($^*$ are significant). Reference values for P-E, R and R$_G$ are taken from Winkelbauer et al. (2022), while OVT and OIT are calculated using the GREP reanalyses (1993–2014) and ArcGate (2005–2010, denoted by $^A$). OVT and OIT are defined as positive northward. Reference uncertainties (±) are taken from Winkelbauer et al. (2022) or calculated from the spread of the GREP ensemble

#### Long term mean seasonal cycles

**Fig. 3:**
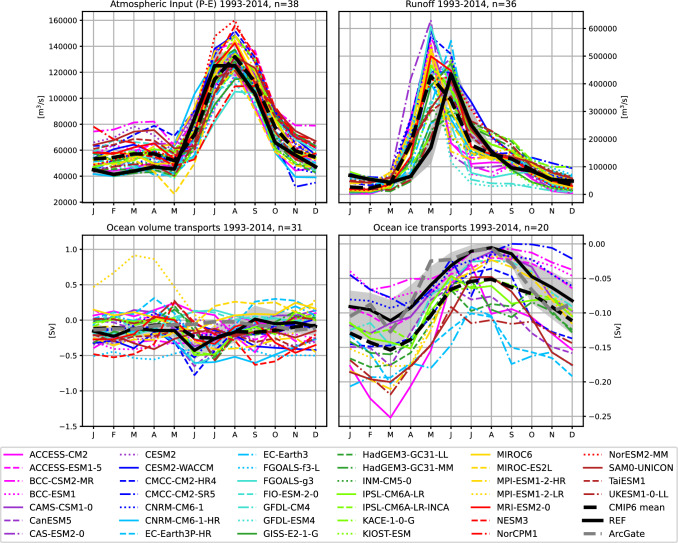
Mean annual cycles of key terms of the oceanic Arctic water budget: **a** atmospheric freshwater input into the ocean (P-E), **b** runoff from Arctic lands (R), **c** oceanic volume transports and (d) oceanic ice transports across the main Arctic gateways. Ice exports from the ArcGate project are based on the PIOMAS reanalysis and not direct measurements. Shading indicates the uncertainty range of the reference values and is either adopted from Winkelbauer et al. (2022) (top panels) or calculated from the spread (2$\sigma$) of the GREP ensemble (bottom panels)

Figure 3 shows the seasonal cycles of the main components of the Arctic water budget. Reference values (Winkelbauer et al. 2022) indicate a peak in net atmospheric freshwater input to the Arctic Ocean (P-E, Fig. 3a) from July to September and input minima during the cold season. The CMIP6 ensemble shows a large spread throughout the year. Most models are able to simulate the timing of P-E peaks and minima correctly, but tend to overestimate net P-E for most of the year (see also Table 1).

The annual cycles of terrestrial runoff are summarized in Fig. 3b). Observations show a strong runoff peak in June, mainly due to snowmelt and river ice break-up, and weak runoff during winter. CMIP6 models disagree on the timing of the runoff peak, with about two-thirds placing the runoff maximum in May. However, while observations are derived from gauge measurements at river mouths, the discharge estimates for CMIP6 are determined by calculating area integrals of runoff at each individual grid point over the whole Arctic catchment. As we do not use any kind of river routing this may introduce an error in the runoff phase - especially for large catchments, routing can lead to delays of several months (Gosling and Arnell 2011). Hou et al. (2023) feed daily runoff outputs from 12 CMIP6 models into a state-of-the-art global river routing model to obtain discharge estimates at river gauges and compare the results with streamflow observations. In general, they find that models tend to perform better in non-cold regions than in cold environments. They find an early bias in the timing of the simulated maximum discharge for cold regions in most of the CMIP6 models evaluated. Therefore, in addition to differences in river routing, differences in runoff phase are most likely related to the ability of models to accurately simulate cryospheric hydrological processes such as snow and permafrost. Gosling and Arnell (2011) find that especially for catchments where the peak flow is strongly influenced by seasonal snowmelt (e.g. Ob and Mackenzie), models tend to overestimate the magnitude of the peak flow and show an early bias of the seasonal peak flows. Kouki et al. (2022) analyse the seasonal snow cover for 33 CMIP6 models and find that the models generally overestimate the spring snowmelt rate, leading to early snowmelt. In addition, they found that the snow water equivalent is generally overestimated in winter, driven by precipitation biases and that, while temperature and precipitation can partly explain the biases in snowmelt, there may also be other contributing factors like inaccuracies in model parameterizations related to snow and the surface energy budget. The shift in the runoff phase also has implications for the seasonal cycles of terrestrial water storage. Wu et al. (2021) assess the annual cycles of terrestrial water storage for 25 CMIP6 models and find a shift in the phase of water storage for the four largest Arctic river basins compared to GRACE satellite data, with an earlier end of the recharge period and an earlier start of the discharge period, which is consistent with our results. Nevertheless, some models appear to get the timing of the runoff peak right (Fig. 3b), however whether this is caused by an actual better representation of the cryospheric processes therein or whether they get the phase right for the wrong reason is not clear and would need further examination.

Figure 3c shows the seasonal cycles of oceanic volume transports through the main Arctic gateways. The GREP ocean reanalysis mean resembles the seasonal cycles of freshwater input to the ocean surface and shows an export maximum of 430 mSv in June, an almost instantaneous response of the ocean to surface freshwater input, as the ocean achieves mass adjustment within about a week through the generation of barotropic waves (Bacon et al. 2015). The observational ArcGate estimate does not show this peak in June, most likely because the mooring arrays are too sparse and the velocity field is not measured accurately enough (both in space and time) to resolve barotropic waves. Models using a non-linear free surface scheme (NLFS), where freshwater from sea ice melt is physically dumped into the ocean resulting in barotropic waves (Madec 2016; Roullet and Madec 2000), were corrected by subtracting the seasonal change in sea ice volume. Volume transports without the sea ice volume correction are shown in the Supplementary material (Fig. S1). The FOAMv2 reanalysis, which in contrast to the GREP ensemble also uses NLFS, as well as CMIP6 models with NLFS show much stronger amplitudes and summer peaks up to one order of magnitude larger than the other models and reanalyses. The effect of ice formation and growth on volume transport can be seen in the cold season, as freshwater is removed from the ocean, leading to a net import of water into the Arctic. However, this behaviour is physically not realistic, as in reality sea ice melt and formation should not affect volume transports in and out of the Arctic. While the correction for net Arctic volume transports appears to be relatively straightforward, the correction for individual Arctic straits and heat transports is not as straightforward and is beyond the scope of this study. Unsurprisingly, the effect of the model mass adjustment of 2-3 Sv in summer is also visible to some extent in the heat transports and will be discussed in Sect. 4.2.1. Meanwhile, linear free surface models show smoother cycles with smaller amplitudes as salt is removed from the ocean during ice melt and added during ice growth to simulate brine rejection. After the correction (Fig. 3c) the seasonal cycles of the NLFS models appear smoother and the CMIP6 MMM stays within uncertainty bounds of our reference estimates during 9 out of 12 months. However, the inter-model spread is still large and there are also models showing some spurious patterns. For example, the MPI-ESM1-2-LR model features volume inflows of up to 900 mSv in spring. As mentioned above, due to the sensitivity of volume transports some errors may be a result of inaccuracies in the calculation process related to the ocean bathymetry. We discuss this further in Sect. 5 and Winkelbauer et al. (2023).

Annual cycles of oceanic ice transports are shown in Fig. 3d. Reanalyses (REF) and ArcGate estimates agree on the annual phase of ice export, with a maximum of ice export in March and a minimum from July to September. Of the 20 CMIP6 models used in this study, which provide all the necessary parameters to calculate ice transports, most agree with the observational estimates in terms of the timing of ice discharge, but differ widely in terms of magnitude. Ice export maxima in March range from -250 mSv (ACCESS-CM2) to -70 mSv (BCC-CSM2-MR), with most models overestimating total sea ice export throughout the year.

### Energy budget

In this section we will assess the key components of the coupled energy budget of the Arctic. We will start by looking at the mean state of the Arctic system and the accumulation of energy (enthalpy) in the Arctic ocean-sea ice system (heat content of the Artcic Ocean and the enthalpy due to sea ice melt).

**Fig. 4:**
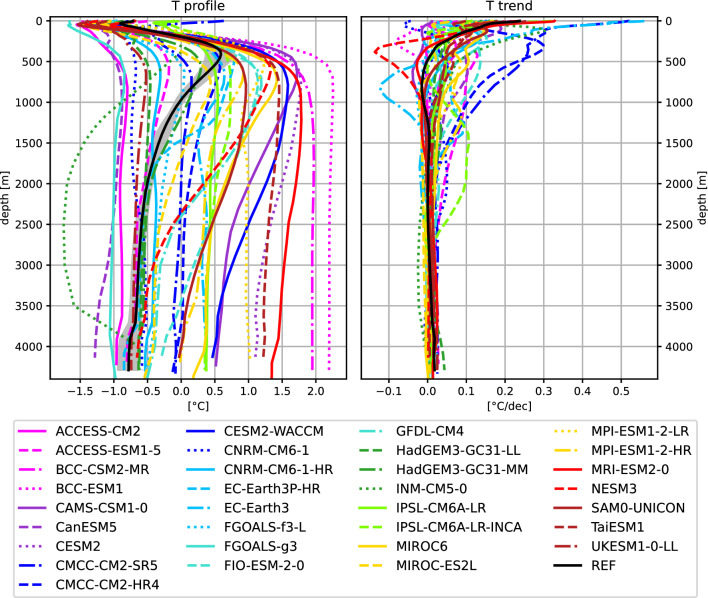
Vetical profiles of area averaged Arctic temperatures and temperature trends over the 1993–2014 period. Reference values are taken from the GREP reanalyses. Shading indicates the spread (2$\sigma$) of the GREP ensemble

Figure 4a shows depth profiles of average Arctic ocean temperatures integrated over our whole study area. The observed halocline lies in the uppermost  250 m, with the warmer and saltier Atlantic Water layer lying underneath. Vertical profiles from the GREP reanalyses (REF) are quite consistent with observed profiles (see e.g. Khosravi et al. 2022) and show an Atlantic water core temperature of about 0.6$-$ 0.8 $^\circ$C and a core depth of about 450 m. Temperature profiles from the CMIP6 models feature substantial biases, especially so in depths below  500 m. Consistent with Khosravi et al. (2022) and Heuzé et al. (2023), we find that CMIP6 models simulate the Atlantic layer too deep and too thick. Further, CMIP6 models feature a large inter-model spread of more than 3 $^\circ$C for layers underneath the halocline.

**Fig. 5:**
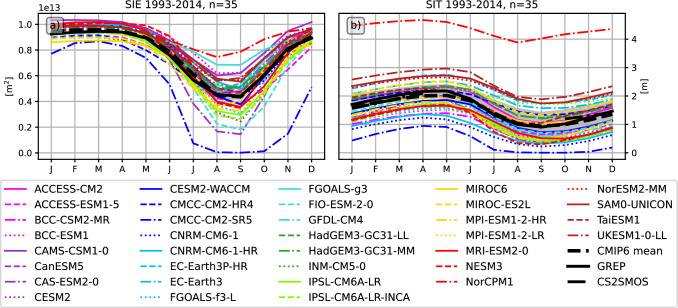
Mean annual cycles of **a** the mean Arctic sea ice extent (SIE) and **b** the mean sea ice thickness for CMIP6 models and the GREP reanalysis. Additionally SIT estimates from CS2SMOS (10-2002–12-2014) are shown. Shading indicates the spread (2$\sigma$) of the GREP ensemble

Figure 5 shows the annual cycles of sea ice extent (SIE) and sea ice thickness (SIT). While the CMIP6 models again feature a large inter-model spread with obvious biases for several models, the MMM actually stays within the uncertainty bounds of our reference estimates for both SIE and SIT.

**Fig. 6:**
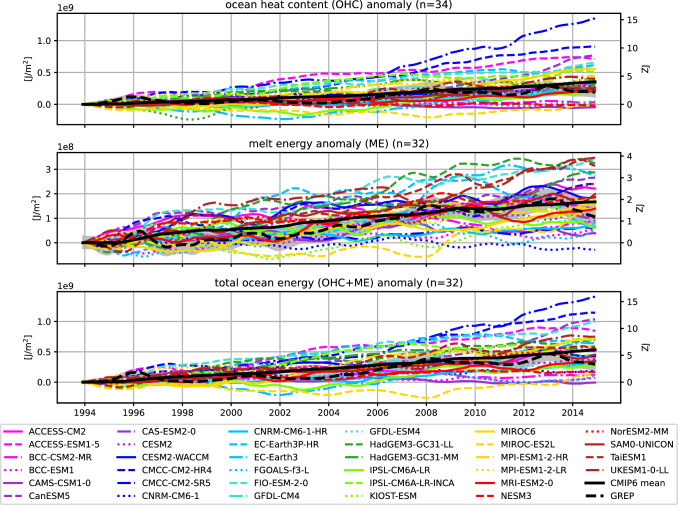
Full-depth anomalous OHC (top) and ME (middle) accumulation as well as their sum (bottom) in the Arctic Ocean since 1993. Number of available CMIP6 models is given in the titles (n). Left axis shows area-averaged changes in J/$\hbox {m}^2$ and the right axis shows area integrated changes in ZJ using a conversion factor of 11.3 $\times$ 10$^{12}$ $\hbox {m}^2$. Shading indicates the spread (2$\sigma$) of the GREP ensemble

Figure 6 shows the heat accumulation in the Arctic since 1993. The starting year 1993 was chosen because of the availability of our observational reference values. The top panel shows the increase in heat contained in the Arctic Ocean at full depth (OHC), as defined in Fig. 1. Ocean reanalyses show an increase of 0.2 GJ/$\hbox {m}^2$ (area integrated values may be calculated using the Arctic Ocean area of 11.3 $\times$ 10$^{12}$ $\hbox {m}^2$ and are provided on the right axes of Fig. 6). While most CMIP6 models agree on an increase in oceanic heat over the period 1993–2014, the amount of heat accumulation varies widely between models. Most models overestimate the heat accumulation, with CMCC-CM2-SR5 being the most extreme with an accumulation of 1.3 GJ/$\hbox {m}^2$. The MMM of all 34 models is almost twice as high as the observational estimate, reaching 0.35 GJ/$\hbox {m}^2$ for the 22-year period. Three models (CAMS-CSM1-0, NESM3, MIROC-ES2L) show a slight decrease in oceanic heat storage over the 20-year period and another three models (BCC-ESM1, BCC-CSM2-MR, FGOALS-g3) show insignificantly small heat accumulations, about an order of magnitude smaller than the reference values.

The middle panel shows the accumulation of energy going into sea ice melt (ME). Ocean reanalyses show a heat accumulation of 0.1 GJ/$\hbox {m}^2$ over the 22-year period, about 57% less than the accumulated OHC change. All CMIP6 models (except one CNRM-CM6-1) agree on an increase of the ME over the last decades, but they again show a huge inter-model spread and range from a total accumulation of 0.0 to 0.4 GJ/$\hbox {m}^2$. The MMM of all 32 models (0.2 GJ/$\hbox {m}^2$) is about 30% higher than indicated by the reanalyses, but remains within the uncertainty of the reanalysis ensemble. The total ocean energy accumulation (OHC+ME) is mostly dominated by ocean heat content and is shown in the bottom panel of 6.

For a deeper understanding of the OHC changes we assess the trends of Arctic Ocean temperatures with depth (Fig. 4b). Reanalyses reveal a strong increase of temperatures of about 0.25 $^\circ$C per decade at the surface. Trends become weaker with depth and beneath about 500 m temperature changes become very small. CMIP6 models show quite diverse trends. While most models agree on an temperature increase at the surface, the strength of the trend ranges from close to zero up to an increase more than twice as high as shown by reanalyses. For the layers below the halocline models differ in terms of sign of the trend and trend strength. For the deep ocean all models agree on comparably small temperature changes. However, it has to be noted, that temperature trends are calculated over a 22-year period, a time-frame short enough that variabilities in in-flowing Atlantic Waters may be of importance. Muilwijk et al. (2018) found that variabilities in northward ocean heat transports may impact temperature changes in the deeper Arctic Ocean, with prominent variability on perennial and decadal time scales as well as indicators of variability on multidecadal scales.

**Fig. 7:**
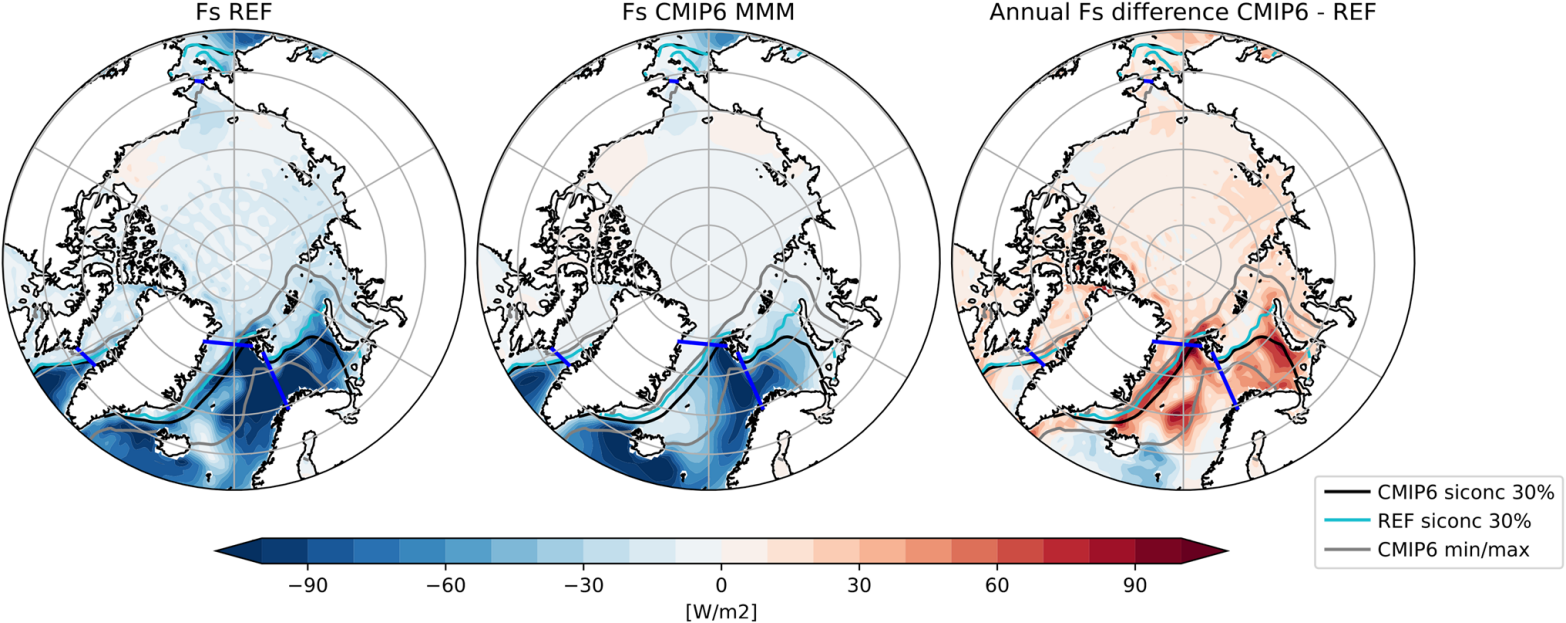
1993–2014 average F$_{S}$ for the observation based estimate (REF, left) and the CMIP6 MMM (middle) as well as their difference (right). 30% sea ice concentration lines are indicated in black, cyan and grey, borders of the study area are marked in blue

Nevertheless, for the 1993–2014 period the large OHC changes simulated by the CMCC-CM2-SR5 model are a result of strong temperature increases from the surface down to about 2000 m depth, while for instance the strong OHC changes for CMCC-CM2-HR4 are mainly driven by temperature changes in the depth of the Atlantic layer core. The NESM3 model, which simulates a slight decrease of heat accumulation, features plausible temperature trends at the surface, however those are compensated by a strong temperature decline around the Atlantic water core depth. For the other five models simulating either insignificantly small heat accumulations or even slight decreases, temperature trends are rather small and partly negative already from the surface down. There are also some other spurious signals to be seen, for instance the EC-Earth3 model simulates net heat accumulations similar to the observed values, however temperature trends at the surface are about twice as high as indicated by our observational reference and in return it features strongly negative temperature trends around 800 m depth. To assess the spurious trends more closely we looked at longer time periods and found some dubious jumps in the models’ OHC time series and partly even changes in the sign of temperature trends when viewing other 22 year periods (not shown). This may indicate that equilibrium was not yet reached by the models and longer spin-up times may be required. We found no clear connection between temperature biases and the strength of temperature trends. We additionally calculated some of the water and energy budget variables for a selection of intra-ensembles containing multiple members of the same models. While intra-model spreads are small for most variables, we found rather large ranges for OHC anomalies, with additionally strong variation from model to model, indicating model-dependence of simulated internal variability. For instance, OHC anomalies at the end of 2014 for an ensemble of 11 CMCC-CM2-SR5 models range between 0.59 and 1.31 $\hbox {GJm}^{-2}$ and for 11 CESM2 models only between 0.19 and 0.29 $\hbox {GJm}^{-2}$. The larger intra-model errors could again be a sign of internal variability or possible spin-up effects. Nevertheless, as errors estimated via our bootstrapping approach are of a similar or even higher value than those estimated from the model ensembles, we believe our uncertainty estimation to be valid.

The OHC and ME accumulations are converted into tendencies following Mayer et al. (2019) using the Theil-Sen trend estimator. Mean rates for 1993–2014 are given in Table 5. Reanalyses indicate a total ocean warming rate (OHCT+MET) of 0.4 $\hbox {Wm}^{-2}$ for 1993–2014, of which about 40% is due to sea ice melting. The CMIP6 MMM shows a total warming rate of 0.7 $\hbox {Wm}^{-2}$, of which about one third is due to MET. The atmospheric warming rate (AET) is more than one order of magnitude smaller than OHCT and MET. The CMIP6 models range between $-$ 0.1 and 0.1 $\hbox {Wm}^{-2}$ (the exception being FGOALS-g3), with an MMM of 0.0 $\hbox {Wm}^{-2}$. Our reference estimate (ERA5) reaches 0.1±0.9 $\hbox {Wm}^{-2}$, while the estimate from Mayer et al. (2019) suggests $-$ 0.1 $\hbox {Wm}^{-2}$. As the latter was calculated only over the 2005–2009 period it may be affected by natural variability on various time scales, as AET is assumed to be positive but close to zero on longer time scales (von Schuckmann et al. 2020).

**Table 5:** Long-term averages for major energy budget components. The MMM is calculated using all available models and REF denotes the observation based reference values. Averaging periods are 1993–2014 for the MMM and REF and 2005–2009 for the Mayer et al. (2019) estimate. Reference values for OHT are taken from reanalyses and ArcGate (2005–2010, denoted by $^A$). Oceanic transports are given in TW and may be converted to $\hbox {Wm}^{-2}$ using an integration area of $11.3\times 10^{12}$ $\hbox {m}^2$. Reference uncertainties (±) are based on the standard deviations of monthly mean values (F$_{S}$, F$_{TOA}$, AET) or calculated from the spread of the GREP ensemble (OHCT, MET, OHT)

	F$_{S}$	F$_{TOA}$	AET	OHCT	MET	OHT
Units	[$\hbox {Wm}^{-2}$]					[TW]
ACCESS-CM2	$-$ 7.1	$-$ 109.3	0.1	0.4	0.3	87.2
ACCESS-ESM1-5	$-$ 9.7	$-$ 111.7	0.0	1.0	0.2	134.8
BCC-CSM2-MR	$-$ 2.8	$-$ 114.0	0.0	0.0	0.1	27.6
BCC-ESM1	$-$ 3.5	$-$ 112.5	$-$ 0.0	$-$ 0.0	0.1	23.6
CAMS-CSM1-0	$-$ 3.5	$-$ 110.0	0.0	$-$ 0.1	0.0	35.6
CanESM5	$-$ 8.0	$-$ 109.8	0.0	1.1	0.4	92.2
CAS-ESM2-0	0.1	$-$ 103.6	$-$ 0.0	-	0.2	–
CESM2	$-$ 6.3	$-$ 115.6	$-$ 0.1	0.4	0.2	68.0
CESM2-WACCM	$-$ 5.9	$-$ 118.4	0.0	0.3	0.3	60.4
CMCC-CM2-HR4	$-$ 14.6	$-$ 114.7	0.0	1.3	0.3	189.8
CMCC-CM2-SR5	$-$ 9.8	$-$ 98.0	0.0	1.9	0.1	144.6
CNRM-CM6-1	$-$ 8.1	$-$ 115.6	0.0	0.3	$-$ 0.0	100.7
CNRM-CM6-1-HR	$-$ 7.8	$-$ 117.1	$-$ 0.0	0.3	0.0	113.5
EC-Earth3P-HR	$-$ 9.5	$-$ 112.0	0.0	0.8	0.2	115.5
EC-Earth3	$-$ 9.8	$-$ 112.8	$-$ 0.0	0.6	0.3	103.2
FGOALS-f3-L	$-$ 5.3	$-$ 111.9	$-$ 0.0	0.1	0.1	64.4
FGOALS-g3	$-$ 5.4	$-$ 111.7	0.2	0.3	-	31.8
FIO-ESM2-0	$-$ 7.4	$-$ 104.7	$-$ 0.0	0.9	0.6	106.0
GFDL-CM4	$-$ 8.7	$-$ 112.9	$-$ 0.1	0.4	0.2	121.9
GFDL-ESM4	$-$ 7.7	$-$ 114.0	0.1	0.3	0.1	–
GISS-E2-1-G	$-$ 2.6	$-$ 113.3	$-$ 0.0	–	–	–
HadGEM3-GC31-LL	$-$ 9.9	$-$ 108.8	0.0	0.6	0.4	115.3
HadGEM3-GC31-MM	$-$ 9.8	$-$ 110.9	$-$ 0.0	0.4	0.4	122.5
INM-CM5-0	$-$ 10.1	$-$ 103.6	0.0	0.6	-	-
IPSL-CM6A-LR	$-$ 8.7	$-$ 114.9	0.0	0.4	0.2	110.6
IPSL-CM6A-LR-INCA	$-$ 8.0	$-$ 115.5	0.0	0.8	0.3	109.5
KACE-1-0-G	$-$ 12.8	$-$ 107.9	0.0	–	–	–
KIOST-ESM	-	$-$ 115.2	-	-	0.2	-
MIROC-ES2L	$-$ 6.7	$-$ 100.4	0.1	$-$ 0.0	0.2	94.1
MIROC6	$-$ 8.0	$-$ 109.6	0.0	0.8	0.3	119.3
MPI-ESM1-2-HR	$-$ 8.4	$-$ 115.7	0.1	0.6	0.2	102.4
MPI-ESM1-2-LR	$-$ 10.9	$-$ 115.3	0.0	0.6	0.2	124.3
MRI-ESM2-0	$-$ 6.1	$-$ 115.2	0.1	0.4	0.3	83.9
NESM3	$-$ 4.7	$-$ 112.6	0.0	$-$ 0.1	0.3	40.0
NorCPM1	$-$ 8.3	$-$ 111.9	$-$ 0.1	-	0.2	-
NorESM2-MM	$-$ 8.6	$-$ 116.3	0.0	0.4	0.4	-
SAM0-UNICON	$-$ 7.0	$-$ 111.8	$-$ 0.1	0.3	0.3	61.8
TaiESM1	$-$ 7.9	$-$ 113.0	0.1	0.3	0.5	78.8
UKESM1-0-LL	$-$ 8.3	$-$ 109.7	$-$ 0.0	0.5	0.6	93.0
**MMM**	$-$ 7.6	$-$ 111.6	0.0	0.5	0.2	92.9
**REF**	$-$ 18.0 ± 3.9	$-$ 116.7 ± 0.5	0.1 ± 0.9	0.3 ± 0.4	0.3 ± 0.1	126.7 ± 3.7 / 151.4$^A$
Mayer et al. (2019)	$-$ 16.2	$-$ 115.8	$-$ 0.1	0.3	0.4	175.2

Table 5 also shows long-term averages of vertical and lateral energy fluxes into the Arctic and results suggest strong biases in several energy budget components. Satellite observations show a net radiation at TOA of $-$ 116.7±1.2 $\hbox {Wm}^{-2}$ for the period 1993–2014 and for the area of interest. Most CMIP6 models show smaller fluxes, the whole ensemble ranging from $-$ 118.4 to $-$ 98.0 $\hbox {Wm}^{-2}$, with a MMM of $-$ 111.6 $\hbox {Wm}^{-2}$.

The net vertical energy flux at the ocean surface (F$_{S}$) from Mayer et al. (2021a) is $-$ 18.0±2.1 $\hbox {Wm}^{-2}$ for the period 1993–2014, while Mayer et al. (2019) estimate a flux of $-$ 16.2 $\hbox {Wm}^{-2}$ for 2005–2009. All CMIP6 models strongly underestimate the outgoing energy fluxes at the surface, ranging from $-$ 14.6 to $-$ 2.6 $\hbox {Wm}^{-2}$, with one model (CAS-ESM2-0) even showing a slightly positive annual F$_{S}$ of 0.1 $\hbox {Wm}^{-2}$. Geographical maps of F$_{S}$ for the individual models are not shown, but it should be noted that all models are able to simulate reasonable large-scale patterns, with low F$_{S}$ values over sea ice and high values from the ocean in the Nordic Seas. However, net F$_{S}$ in the Nordic Seas shows even larger biases with the CMIP6 MMM being about 30% lower than indicated by our reference (not shown). Figure 7 shows the long-term averaged F$_{S}$ for our observational estimate (left panel), the CMIP6 MMM (middle panel) and their difference (right panel). Furthermore, 30 % sea ice concentration isolines are shown. Differences in F$_{S}$ over the central, sea ice covered Arctic are small, with slightly higher values around the Kara, Laptev and Chukchi Seas. The largest differences in F$_{S}$ occur near the sea ice edge between Greenland and Svalbard, in the Barents Sea and in the Norwegian Sea in proximity of the Lofoten Basin, with differences of up to 80 $\hbox {Wm}^{-2}$. The exact position of the sea ice edge in the Nordic Seas varies considerably between CMIP6 models (indicated by the grey lines in Fig. 7), with the MMM sea ice edge being positioned further south than the reference. Thus, most CMIP6 models simulate too little open water, resulting in smaller net outgoing energy fluxes. For the Labrador Sea and the Bering Sea, the sea ice concentration lines between our reference and CMIP6 are in good agreement and the differences in F$_{S}$ are comparatively small. Apart from sea ice, the sea surface temperature has major effects on F$_{S}$. For example, F$_{S}$ biases in Lofoten Basin are mainly caused by regional cold biases in the simulated sea surface temperatures (not shown).

The loss of energy to space over the Arctic is balanced by northward heat transports in atmosphere and ocean. The CMIP6 models show an enormous range of simulated oceanic heat transports, ranging from 20.30 to 189.82 TW (corresponding to a convergence of 1.80 to 18.80 $\hbox {Wm}^{-2}$), with a MMM of 93.3 TW (8.26 $\hbox {Wm}^{-2}$). For the same period, reanalyses indicate a long-term average heat flux of 126.7 TW for 1993–2014. Observational estimates (Tsubouchi et al. 2012) are only available for the period 10/2004-05/2010, but they show an even higher heat transport of 151.4 TW (13.40 $\hbox {Wm}^{-2}$). For the same period, the reanalysis is 136.2 TW (12.05 $\hbox {Wm}^{-2}$) and the CMIP6 MMM is 98.1 TW (8.68 $\hbox {Wm}^{-2}$), clearly underestimating the lateral energy input. Table 5 shows that while most models underestimate the reference value, there are 6 models in particular (BCC-CSM2-MR, BCC-ESM1, CAMS-CSM1-0, FGOALS-g3, FGOALS-f3-L and NESM3) that have exceptionally low transports, with values more than 50% lower than our reference estimates. Some of these use the same ocean model component, BCC-CSM2-MR, BCC-ESM1 and CAMS-CSM1-0 use MOM4, while FGOALS-g3 and FGOALS-f3-L use LICOM3.0. Therefore, it would be a useful step to scale the models in terms of their independence through appropriate weighting algorithms to obtain reliable MMM.

**Fig. 8:**
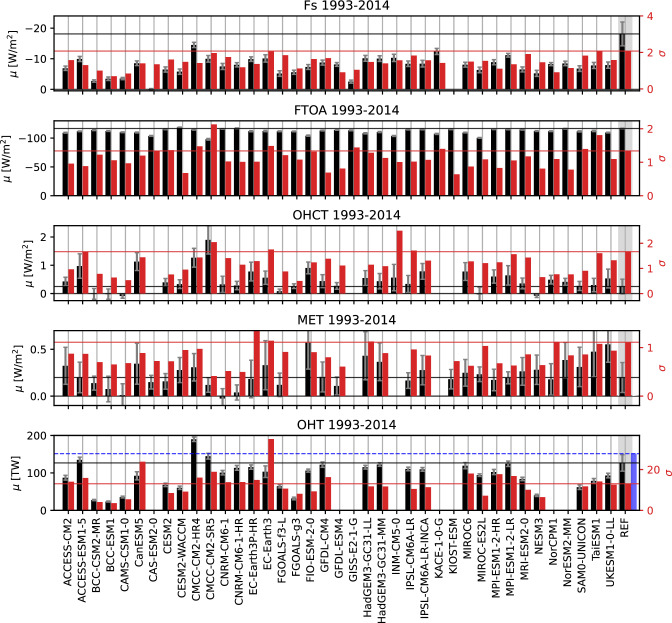
Averages (black, left axis) and standard deviations of annual averages (red, right axis) for the major Arctic energy budget components. Reference values (REF) are indicated by horizontal lines and shown on the right hand side of the panels. They are taken from Mayer et al. (2021a), DEEPC, ERA5 and the GREP reanalyses (1993–2014). Additionally also transports from the ArcGate project (2005–2010) are shown (blue bars and dashed lines). Error bars denote sampling errors for CMIP6 and errors calculated from the spread of used observational data for REF

Figure 8 shows long-term averages of the major energy fluxes and tendencies for all models and the reference-based estimates, whereby especially the models’ biases in Fs and OHT stand out. Additionally, standard deviations of annual averages and sampling errors are shown. The large sampling errors for OHCT and MET highlight the high temporal variabilities in those variables, which, as discussed above, may indicate possible residual spin-up effects.

#### Long term mean seasonal cycles

**Fig. 9:**
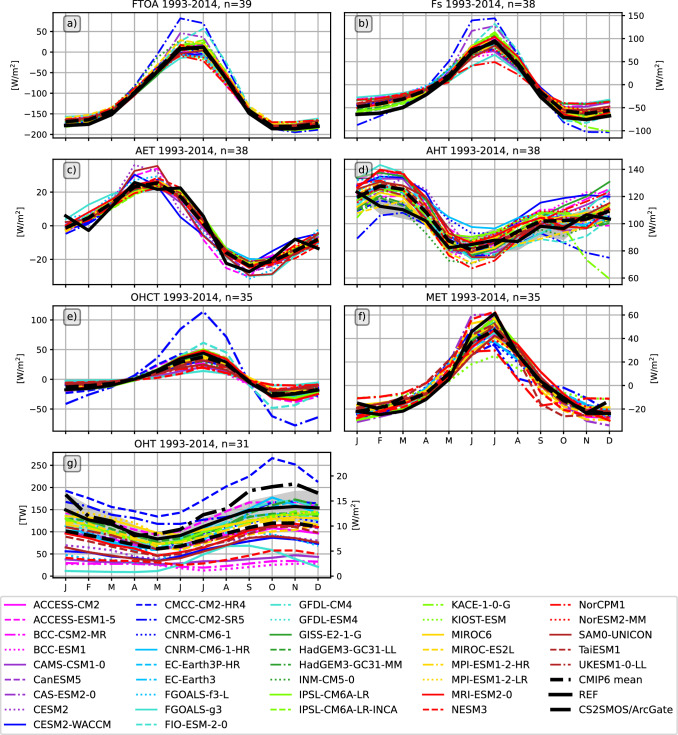
Mean annual cycles of the key terms of the coupled Arctic energy budget: **a** net radiation at the top of the atmosphere F$_{TOA}$, **b** net vertical energy flux at the surface F$_{S}$, **c** atmospheric energy tendency AET, **d** atmospheric heat transport AHT, **e** full-depth ocean heat content tendency, **f** melt energy tendency (MET), and **g** the oceanic heat transport across the main Arctic gateways. Shading indicates the uncertainty range of the reference values and is either based on the 2$\sigma$ standard deviations of monthly mean values (F$_S$, F$_{TOA}$, AET, AHT) or calculated from the spread of the GREP ensemble (OHCT, MET, OHT)

Figure 9 shows the mean annual cycles of the main energy budget terms in Eqs. 1 and 2. Averaging periods depend on the availability of reference data and are indicated in the figure titles. In general, most models are able to simulate the general shape of the annual cycles accurately, but there are also some obvious biases and differences, which are discussed in more detail below.

**Fig. 10:**
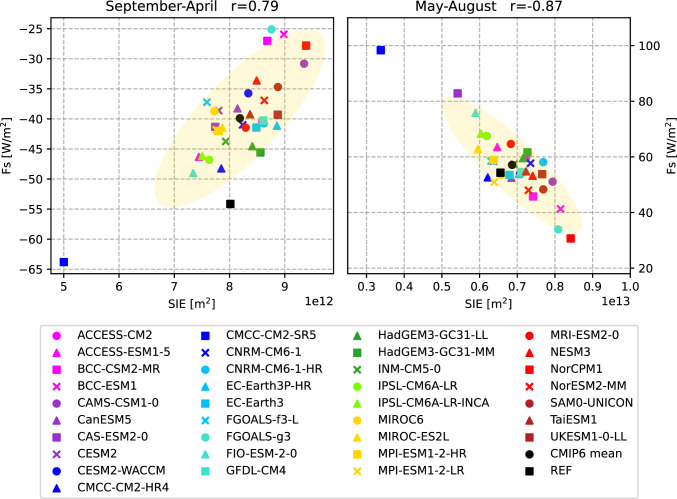
Scatter plots between the net surface flux F$_{S}$ and mean sea ice extent SIE averaged over 1993–2014. Left panel: September-April correlation, right panel: May–August correlation. Yellow ellipses show the 2-sigma confidence ellipses for the CMIP6 models

The net radiation at TOA is shown in Fig. 9 a. It is strongly negative for most of the year and only slightly positive in June and July. This strong seasonal cycle is mainly driven by solar radiation. The spread (max-min) between CMIP6 models is relatively small in winter and the transition seasons, reaching a maximum of 95 $\hbox {Wm}^{-2}$ in summer. A few models reach unrealistically high values during summer, in particular the CMCC-CM2-SR5 model shows a maximum of more than 80 $\hbox {Wm}^{-2}$ (compared to 12 $\hbox {Wm}^{-2}$ from observations), mainly due to strongly underestimated reflected shortwave radiation as a consequence of low sea ice biases (Fig. S2 in Supplementary material). About 20% of the models (8 out of 39) simulate negative F$_{TOA}$ throughout the year. Inter-model spread is higher during summer, nevertheless the CMIP6 MMM is in quite good agreement with the observational estimate (DEEPC) and stays within the observational uncertainty bounds during those months. In winter, the inter-model spread is smaller, but most models underestimate the strong winter minima and the MMM is up to 10 $\hbox {Wm}^{-2}$ lower than indicated by observations. The net radiation at TOA is an important driver of the annual cycle of the surface energy flux F$_{S}$, so F$_{S}$ shows a similarly strong annual cycle. F$_{S}$ remains negative (outgoing) during winter, and with the maximum of incoming shortwave radiation in May, F$_{S}$ becomes positive and reaches its maximum in summer as sea ice melt progresses. Similar to F$_{TOA}$, some models have unrealistically high summer maxima (CMCC-CM2-SR5, CAS-ESM2-0, FIO-ESM-2-0), caused by underestimated reflected shortwave radiation due to too little sea ice (not shown). The CMCC-CM2-SR5 model strongly underestimates both the total extent of sea ice in the area of interest and the thickness of the sea ice cover (Fig. 5), and in the summer months CMCC-CM2-SR5 even simulates an ice-free Arctic. CAS-ESM2-0 and FIO-ESM-2-0 are also at the lower end of the SIE ensemble, while models with high SIE during the summer months (e.g. NorCPM1, FGOALS-g3) also simulate low F$_{S}$ during the summer months. In winter, most models simulate lower net upward F$_{S}$ than our reference estimate (inferred F$_{S}$, Mayer et al. 2021a). However, CMCC-CM2-SR5 overestimates the winter minima because the SIE is quite small in winter, leading to unrealistically strong outgoing longwave radiation and latent heat fluxes (not shown). Figure 10 shows scatter plots between long-term average F$_{S}$ and SIE. The correlations are divided into the season with negative net F$_{S}$ (September - April) and the season with positive net F$_{S}$ (May - August). The correlations are high throughout the year. In summer, when incoming solar radiation is high, models with little sea ice simulate higher incoming net radiations, mostly caused by reduced reflected shortwave radiations (not shown). In autumn and winter, when the incoming solar radiation is low to non-existent, models with less sea ice simulate higher outgoing longwave radiations (not shown) and therefore lead to higher negative net radiations.

Figure 9c shows that the annual cycle of the atmospheric energy storage component AET is moderate compared to the other atmospheric components, and that CMIP6 models reproduce the observed cycles (ERA5) quite well. Atmospheric energy transport (AHT) for CMIP6 is estimated as residual using Eq. 1. Inter-model spread is relatively high throughout the year with most CMIP6 models simulating higher transports than indicated by our observational reference (ERA5). Biases are strongest from late autumn to early spring and are connected to biases in surface energy fluxes and therefore biases in the position of the sea ice edge as well as sea surface temperatures. However, in summer, where biases in F$_S$ and F$_{TOA}$ are at their peaks in some models (e.g., CMCC-CM2-SR5, CAS-ESM2-0 and NorCPM1), compensating effects lead to smaller biases in AHT. Meanwhile, biases in AET play a less prominent role and adjust the total AHT biases with smaller reinforcing and compensating effects.

Figure 9e shows the annual cycles of the oceanic storage component OHCT. The models agree on ocean warming in the summer months and ocean cooling in the winter, but the amplitude of the cycles varies considerably. The model scatter is large for most of the year, with 95% of the models within 12–43 $\hbox {Wm}^{-2}$ in summer and − 24 and − 4 $\hbox {Wm}^{-2}$ in winter. The most obvious exception is CMCC-CM2-SR5, which has summer maxima about three times higher than the MMM and winter minima about three times lower. These large variations are again closely related to the underestimation of sea ice in CMCC-CM2-SR5. An amplification of the OHCT seasonal cycle with declining sea ice is expected, and in fact has already been observed over recent decades (Mayer et al. 2016), but of course to a much lesser degree compared to CMCC-CM2-SR5. The annual cycles of the melt tendency (MET) are shown in figure 9f. The reference values are calculated from the GREP ensemble. The majority of models simulate the phase of the annual MET cycle correctly, but the inter-model spread is large throughout the year. Winter values range from − 31 to − 10 $\hbox {Wm}^{-2}$ and summer peaks are between 16 and 52 $\hbox {Wm}^{-2}$. The MMM amplitude is generally lower than the reference estimate, with weaker freezing in late winter and early spring and weaker melting during the summer months.

**Fig. 11:**
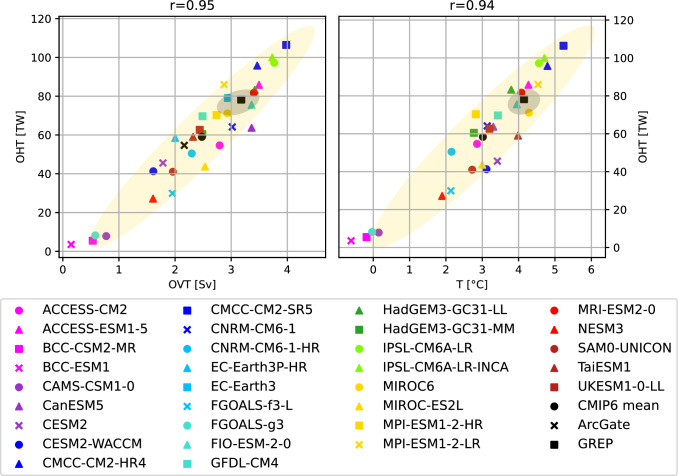
Barents Sea Opening correlations of long-term annual averaged ocean heat transports (OHT) and ocean volume transports (OVT, left panel) as well as BSO average temperatures (right panel) for various CMIP6 models (1993–2014), the GREP reanalyses mean (1993–2014) and ArcGate observations (2005–2010). Positive values denote transports into the Arctic. Yellow ellipses show the 2-sigma confidence ellipses for the CMIP6 models and grey ellipses for the GREP reanalyses

The annual cycles of the net oceanic heat transports are shown in Fig. 9g. The large inter-model variability is evident throughout the year, but all models agree on an inflow of heat to the Arctic in all calendar months. Most models are able to simulate the timing of the inflow extremes correctly, with a minimum in May and a maximum in late autumn and early winter. Reference values (REF) are derived from the GREP ocean reanalysis ensemble, and the observational annual cycle from ArcGate is also shown. Almost all models underestimate the heat influx compared to REF and ArcGate. Only CMCC-CM2-HR4 simulates larger heat transports throughout the year than indicated by observations, with an October peak about 25% higher than the ArcGate estimate of about 200 TW. In addition, CMCC-CM2-SR5, MPI-ESM1-2-HR and MPI-ESM1-2-LR exceed observations in spring. The heat transports for BCC-CSM2-MR, BCC-ESM1, CAMS-CSM1-0, FGOALS-g3, FGOALS-f3-L and NESM3 are too low in all calendar months and are mainly caused by biases in the inflow of Atlantic waters through the Barents Sea opening. Heat transports through the individual Arctic straits are shown in the Supplementary material (Fig. S3). In general, while most models are able to simulate the shape of the annual transport cycles to some extent, the inter-model spread is large for all Arctic straits. Seasonal cycles for the BSO feature similar spreads and biases as the net Arctic heat inflow, reflecting the leading role of BSO in determining the amount of oceanic heat entering the central Arctic. Figure 11 shows correlations between BSO heat transports with BSO volume transports and BSO average ocean temperatures. Correlations are high both for volume transports and temperatures indicating biases in the simulated temperatures and currents. It is worth noting that volume transports and strait average temperatures are not independent of each other and feature moderate to high correlations (not shown). The models with exceptionally low OHT values show mean temperatures around 0 degrees Celsius or even slightly negative values. Additionally, they simulate Norwegian Coastal Currents (NCs) that are generally too weak, slowed down too far south, or even negative, while high OHT values are driven by high volume transports due to strong NCs and higher temperatures. Figure 4 revealed that some of the models (including BCC-CSM2-MR, BCC-ESM1 and CAMS-CSM1-0) feature large positive temperature biases in and underneath the Atlantic water layer. However, in the upper most layers some of those models show negative biases and underestimate the actual temperatures in the surface and halocline layers. Figure S4 shows temperature profiles averaged along the individual straits. The BSO profile reveals that while the largest temperature biases for BCC-CSM2-MR, BCC-ESM1, CAMS-CSM1-0 and FGOALS-g3 are found at the surface, where three of the models even simulate temperatures below 0$^\circ$C, negative temperature biases are present in all layers of the rather shallow BSO. These low temperatures near the surface are tightly coupled to the overlying sea ice cover. However, the sea ice cover does not only affect heat transport, but the link between oceanic transports and sea ice goes both ways, as increased heat transports also lead to less sea ice (Årthun et al. 2019).

Heat transports through Fram Strait (Fig. S3a) are too small in the majority of models, but it is possible that models with the NLFS scheme are affected by the mass adjustment due to sea ice melt, as the differences in volume transports between models with non-linear and linear surfaces are largest for Fram Strait (not shown), leading to a simulated maximum volume outflow in summer for NLFS models and a minimum about 2 Sv smaller for those without NLFS. Volume transports through Fram Strait are generally biased low in CMIP6 (Heuzé et al. 2023). However, while net transports show a large spread similar to the BSO (Tab. 6), the MMM actually stays well within the uncertainty range of the reference values. Temperature profiles (Fig. S4) at Fram strait show that virtually all models feature a positive temperature bias below 500 m and above that the majority of models features a negative temperature biases. Therefore, the low biases in OHT are mainly caused by warm biased deep waters flowing out of the Arctic through the East Greenlandic Current (EGC) and cold biased waters flowing into the Arctic via the more shallow West Spitsbergen Current (WSC).

The effect of model spatial resolution can be seen for heat transports through Davis Strait (Fig. S3c). All CMIP6 models with a horizontal resolution of 1/4 degree simulate an OHT peak in autumn, similar to the observational ArcGate estimate, while coarser resolution models do not show such a peak. Somewhat surprisingly, the reanalysis-based estimates, which also feature a horizontal resolution of 1/4 degree, do not simulate such a peak, however they are known to have a cold bias in the West Greenland Current (Pietschnig et al. 2017). The high resolution CMIP6 models however feature stronger and warmer West Greenlandic Currents and stronger, but similarly tempered, Baffin Island currents during autumn (see Fig. S5).

**Fig. 12:**
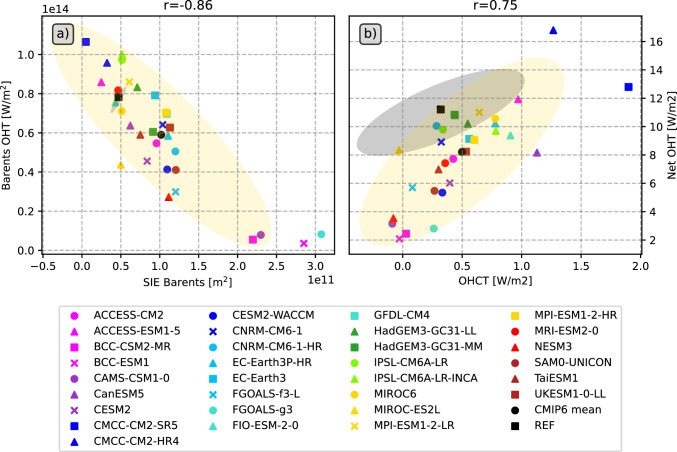
Scatter plots of the effect of ocean heat transports on sea ice and the ocean warming rate. a) correlations between oceanic heat transports through the Barents Sea Opening and the mean sea ice extent in the Barents Sea, b) correlations between net Arctic oceanic heat transports and the oceanic heat content tendency. All values are long-term annual averages over the 1993–2014 period. Yellow ellipses show the 2-sigma confidence ellipses for the CMIP6 models and grey ellipses for the GREP reanalyses

The strength of OHT has important implications for the state of the Arctic Ocean and sea ice. Figure 12 shows scatter plots of OHT, sea ice extent and the ocean warming rate OHCT. As mentioned above there is a tight coupling between sea ice and heat transports. The left panel shows this correlation for the BSO, as models with higher/lower heat transports simulate smaller/larger sea ice areas. This leaves two possibilities: either reduced OHTs allow more sea ice to form, or a larger sea ice cover slows down currents, cools the ocean and therefore leads to lower heat transports. While the effect of OHT on Arctic sea ice has been discussed in various observational (e.g., Årthun et al. 2012; Onarheim and Årthun 2017) and modelling (e.g., Årthun et al. 2019; Dörr et al. 2021) studies, the influences of changes in Arctic sea ice on oceanic circulations, temperatures and therefore heat transports have been less investigated and still pose many unknowns (Docquier and Koenigk 2021). More thorough analysis and model experiments would be required to clarify this possible bidirectional effect, but, this is beyond the scope of this study.

Ocean heat transports also affect the change in oceanic temperature. Figure 12 b) shows the correlation of heat transports and the change in ocean heat content: models with larger/smaller OHT show a faster/slower warming of the Arctic Ocean.

**Fig. 13:**
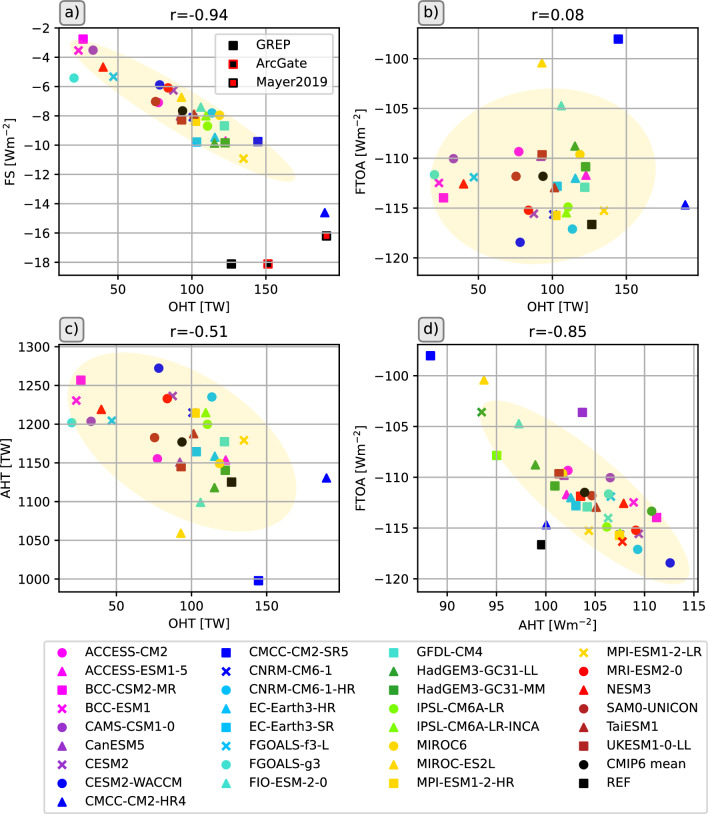
Scatter plots of long-term annual averages of oceanic heat transports and atmospheric energy budget components. Correlations between **a** OHT and the net surface energy flux F$_s$, **b** OHT and the net energy flux at the top of the atmosphere F$_{TOA}$, **c** OHT and atmospheric heat transports AHT, **d** AHT and F$_{TOA}$. Yellow ellipses show the 2-sigma confidence ellipses for the CMIP6 models

The consequences of biases in the oceanic components may also pass over to the Arctic atmosphere, potential effects are shown in Fig. 13. There are strong correlations between simulated long-term averaged OHT and F$_s$ (Fig. 13a), as OHT driven changes of sea ice and ocean temperature strongly affect the reflected shortwave radiation during summer and outgoing longwave radiations as well as turbulent energy fluxes. However, there are no significant correlations between OHT and the net radiation at the top of the atmosphere (Fig. 13b) and biases do not seem to reach up to the top of the atmosphere. In contrast, Fig. 13d shows high correlations between long-term averaged atmospheric heat transports AHT and F$_{TOA}$, as models with weaker outgoing F$_{TOA}$ also feature weaker AHT. OHT and AHT feature moderate anti-correlation (Fig. 13c) and the atmosphere compensates for variances in OHT to the extent that its biases are not seen at the TOA. Models with stronger OHT feature stronger F$_s$ and therefore weaken the atmospheric gradients and subsequently AHT. Note the deviation of the reference values from the model based reference ellipse in 13a. This is caused by inconsistency in our reference based estimates for F$_s$ and OHT and will be discussed futher in the next section.

The impact of OHT on other components of the Arctic system highlights the importance of detecting the exact source of any possible biases therein. To check whether the biases in OHT are also present further south, where less sea ice is present, we calculated transports through the Greenland-Scotland Ridge (GSR, dashed orange line in Fig. 1). Figure S6 shows heat transports at the GSR and figure S7a shows scatter plots between heat transports though the GSR and the sum of transports through Fram Strait and the BSO. They show a high correlation with biases of heat transports being also present further south in the Nordic Seas, tightly coupled to biases in the GSR across strait temperatures (Fig. S7b). Figure S7a shows a group of models slightly to the left of the reference estimates, simulating realistic GSR transports and lower Fram and BSO transports. This shift is actually caused by too much sea ice in the models, which forces the heat out of the ocean in the Nordic Seas through higher outgoing surface energy fluxes.

**Fig. 14:**
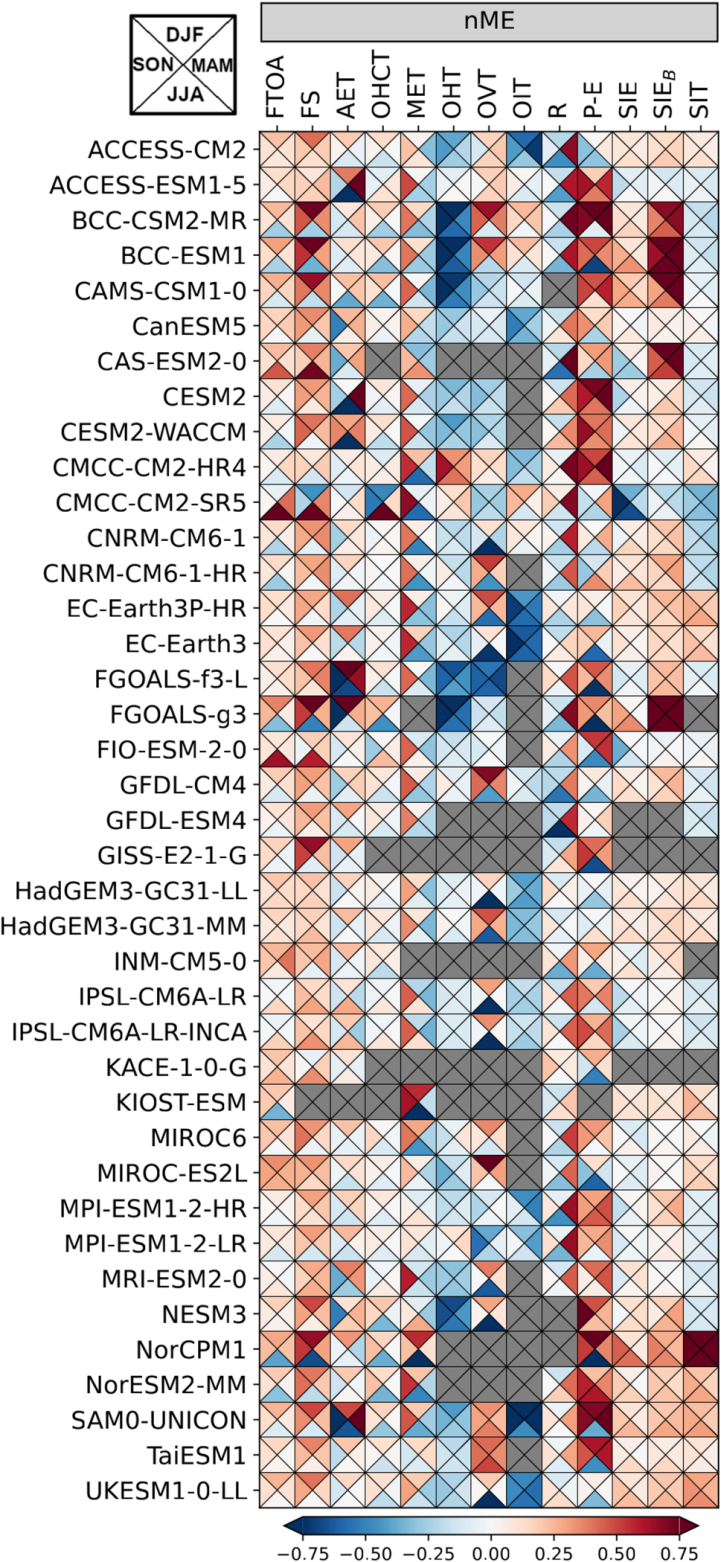
“Portrait” diagram of seasonal normalized mean errors (nME) for various water and energy budget components for 1993–2014. Triangles indicate the respective seasons DJF (upper triangle), MAM (right triangle), JJA (bottom triangle), and SON (left triangle). SIE$_B$ denotes the sea ice extent in the BSO

Figure 14 summarises the seasonal performance of the models and shows normalised mean errors for all models and variables for the energy and water budgets. Seasons are subdivided by triangles, as indicated in the top left-hand corner of the figure. Mean errors for each variable have been normalised by the largest error of the concerning variable to allow for better inter-model comparisons. The closer the values are to 0, the smaller the model bias and the better the model performance. For instance, the net surface energy flux F$_s$ is biased positive from autumn to spring and biased negative in summer for most models, meaning that there is less outgoing energy during the colder seasons and less net incoming energy during summer for those models (see Fig. 9). In contrast, the CMCC-CM2-SR5 model shows a positive bias during summer (more net incoming energy) and a negative bias during winter, caused mainly by its large negative sea ice bias. Further, the connection of biases in sea ice and oceanic heat transports is evident, as models with positive biases in sea ice extent have a negative OHT bias, while biases in sea ice thickness seem to be less relevant with regard to OHT. Seasonal biases in OVT for some models are caused by the models NFL scheme. The affected models are biased negative in summer (stronger outgoing flux due to the sea ice melt effect) and biased positive in winter (effect of ice formation and growth). However, also models without the NFL scheme tend to feature some spurious features. Biases in runoff are mostly due to the one-month shift in the simulated annual cycle, with summer runoff being biased small and spring runoff being biased large. MET biases are largest in the transitional seasons (spring and autumn), while OHCT biases are largest in winter and summer, indicating a weakened amplitude of the annual cycle for most models and an enhanced amplitude for the CMCC-CM2-SR5 model.

### Budget closure

Non-closure of global budgets may contribute to unforced long-term changes/trends in state variables, the so-called model “drift”. This may distort the estimate of forced changes in coupled climate simulations and lead to false interpretations. On a global scale Irving et al. (2021) find non-negligible drift trends in time-integrated ocean heat and freshwater fluxes, F$_{TOA}$ and moisture flux into the atmosphere (evaporation minus precipitation), suggesting a considerable leakage of mass and energy in the simulated climate system. To our knowledge, budget closure on a more regional scale for the Arctic area has not been assessed yet for CMIP6. We use all terms from equations 2 and 3 and ignore changes in the oceanic volume storage, which are considered small (Winkelbauer et al. 2022), to assess the energy and water budget closure for the Arctic Ocean:

8 $$\begin{aligned} Res_{energy}= & {} F_{S} - OHCT - MET - \nabla \cdot F_O - \nabla \cdot F_I + L_f(T_p)P_{snow}\nonumber \\{} & {} - L_f\rho _{snow}\frac{\partial d_{snow}}{\partial t} \end{aligned}$$

9 $$\begin{aligned} Res_{water}= & {} P+ET+R-\nabla \cdot F_{vol} \end{aligned}$$

**Fig. 15:**
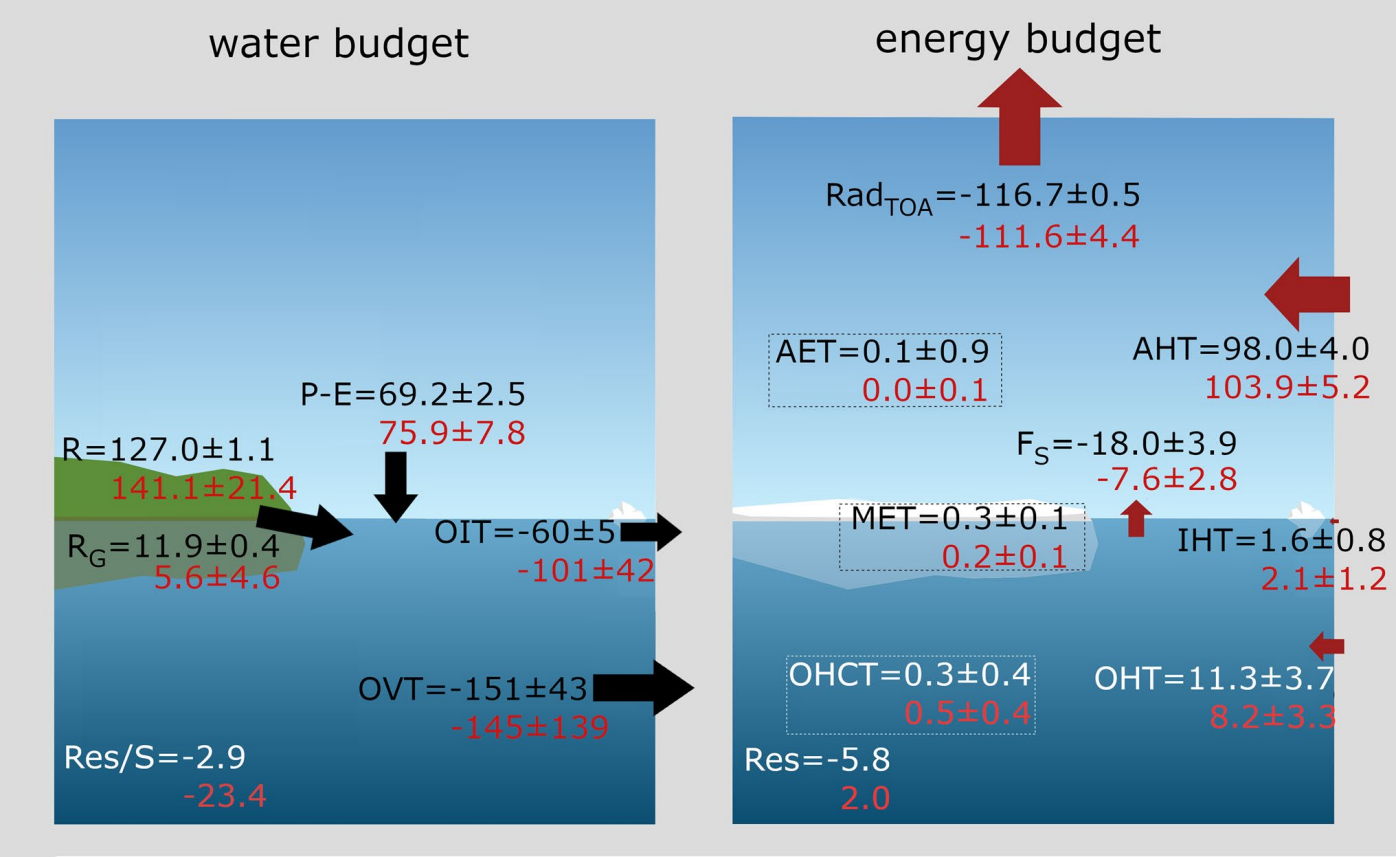
Water (left, in 10$^3$ $\hbox {m}^3$/s) and energy (right, in $\hbox {Wm}^{-2}$) fluxes and storage rates for the reference estimates (black) and the CMIP6 MMM (red) for 1993–2014. Additional estimates from ArcGate and Winkelbauer et al. (2022) as well as Mayer et al. (2019) are given in Table 7. The graphic designs of the schematics are adapted from Winkelbauer et al. (2022) and Mayer et al. (2019)

Annual mean fluxes and storage components for the water and energy budgets as simulated by the CMIP6 MMM (red values) and our reference estimates (black values) are shown in Fig. 15. Note that the reference estimates were taken from multiple independent data sources and are not consistent and therefore the observational budget estimates are not closed but rather feature budget residuals. Comparisons with estimates from Mayer et al. (2019) and Winkelbauer et al. (2022) are given in Table 7.

**Fig. 16:**
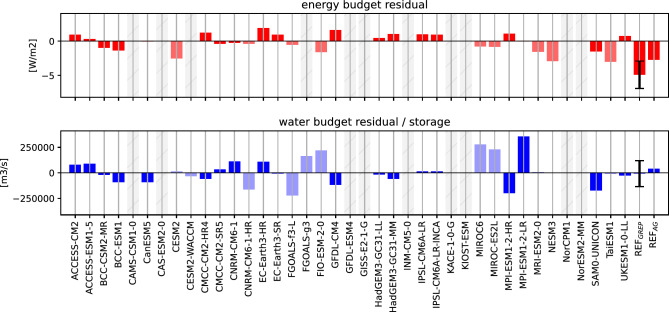
Budget residuals for the energy (top) and water (bottom) budget of the Arctic Ocean

Figure 16 shows residuals for the energy (top) and water (bottom) budgets. With a snowfall term of 1 $\hbox {Wm}^{-2}$ (ERA5) energy budget residuals for the reference estimates using oceanic transports from the GREP ensemble (REF$_{GREP}$) are at $-$ 4.8 $\hbox {Wm}^{-2}$. Residuals using oceanic transports from ArcGate (REF$_{AG}$) are smaller at $-$ 2.6 $\hbox {Wm}^{-2}$. As already seen in Fig. 13a, the largest inconsistencies are found between net surface energy fluxes derived from a combintation of CERES-EBAF TOA fluxes and atmospheric energy budget quantities provided by Mayer et al. (2021a) and oceanic lateral heat transports from the GREP ensemble. Surface energy fluxes as seen by the ocean reanalyses are actually about 3 $\hbox {Wm}^{-2}$ smaller (not shown), explaining the observation based budget residuals. While Mayer et al. (2021a) use ERA5 data, the ocean reanalyses in GREP are not coupled and use atmospheric forcing from ERA-Interim, which already features significantly smaller surface energy fluxes than ERA5 (not shown). Further, the GREP reanalyses calculate their own upwelling fluxes influenced by their own ice thicknesses and skin temperatures, while ERA5 sees constant sea ice thickness of 1.5m and is known to have a warm temperature biases over sea ice (Wang et al. 2019). For the water budget reference based residuals are at $-$ 2.9$\times$10$^3$ $\hbox {m}^3$ (REF$_{GREP}$) and $-$ 52.1$\times$10$^3$ $\hbox {m}^3$ (REF$_{AG}$).

Figure 16 further shows budget residuals for the individual CMIP6 models. Residuals for the energy budget are comparatively small with values between $-$ 2.5 and 2 $\hbox {Wm}^{-2}$. Residuals for the water volume budget are mostly smaller than ± 100$\times$10$^3$ $\hbox {m}^3$. Some models feature larger residuals (e.g. MPI-ESM1-2-LR), however for those models, as discussed above, our volume transports calculations may not be accurate enough.

There are multiple potential reasons for non-closure, some of them are listed below:

- Even though we are confident in our methods of calculation, we still can not preclude problems with our technical analyses. Especially the calculation of Arctic ocean volume transports is very sensitive to the ocean bathymetry and many large fluxes of opposing sign sum up to a relatively small net transport. Therefore, small inaccuracies in the methods of calculation may lead to major errors in net integrated transports. It also has to be noted, that the needed information to calculate exact oceanic volume transports, like exact ocean depths, is not readily available for all models. This will be discussed further in the conclusions section.
- We consider the budget equations as complete as possible, however there is still the possibility that we are missing some smaller budget terms. While small themselves, they still could have effects when trying to close the budgets. For example, oceanic transports are calculated as the sum of the four major gateways, but we neglect transports through the smaller channels of Hecla and Fury Strait. Further, we ignore the small fluxes associated with the change in sensible heat content of ice (IHCT in Eq. 2) and also the temporal (sub-monthly) eddy component of oceanic transports. Also, it’s possible that not all components are provided in the CMIP6 model output, e.g. small terms like numerical diffusion and mass leak increments.
- Imbalances may also arise from deficiencies in the models itself, including model coupling, numerical schemes and/or physical processes. While it is desirable for regional budgets in climate models to be closed, achieving a perfect closure can be challenging. The closure of regional budgets depends on the accuracy and representation of processes within the model, the spatial and temporal resolution of the model, and the quality of parameterizations. However, due to the complexity of Earth’s climate system, including interactions between different components (atmosphere, ocean, land, and ice), achieving complete closure at a regional scale is challenging (Lauritzen et al. 2022).

## Summary and discussion

This study analyses the performance of 39 CMIP6 models in simulating the energy and water budgets of the Arctic. We find systematic biases in several energy and water budget components and large inter-model spreads in most evaluated parameters when compared to the uncertainty of the observationally constrained estimates.

We assessed model performance by comparing historical long-term averages and seasonal cycles of key energy and water cycle components with observational reference data. The main results of this study are summarised below.

Long-term averaged surface freshwater fluxes tend to be overestimated by most of the models analysed, and apart from large model spreads in their seasonal cycles, we also found an early timing bias of one month in the runoff cycle phase, most likely related to the models’ disability to correctly simulate the timing of snow melt and permafrost degradation.

The introduction of the StraitFlux tools Winkelbauer et al. (2023) allowed the calculation of oceanic transports consistent with the discretization schemes of the respective models, allowing a fair comparison and avoiding spurious artifacts that would be caused by interpolation. However, the results of oceanic volume and ice transports show strong biases. Inter-model spread is large and the majority of models fail to simulate the annual cycles of the net Arctic volume transports correctly. The largest errors and some spurious peaks in summer are introduced by the use of the NLFS scheme for sea ice meltwater. Seasonal cycles of volume transports corrected for sea ice volume still show a large spread with some suspicious-looking models, but the MMM of the corrected fluxes is in better agreement with the reference cycles and is within the uncertainty range of the reanalyses during 9 of 12 months. The calculation of volume transports is very sensitive to the exact ocean bathymetry. We found that for the individual Arctic straits biases due to inaccurate handling of the bathymetry are comperatively small and mostly amount to less than 10 %. However, as the individual fluxes sum up to a rather small net Arctic volume flux, those biases may cause some significant errors for the net transports. As discussed in Winkelbauer et al. (2023) caution is advised especially when calculating volume transports for shallow or bathymetrically more complicated straits, where currents are intensified in the proximity of the ocean ground or coast. While the calculation of heat and salinity transports is not as sensitive, it is still not neglectable in most cases. To improve the calculation of transports we would need the exact cell thicknesses either at the positions where the oceanic temperatures and velocities are defined or, if only thicknesses at the middle of the grid cell are supplied, provide the transformation equations to transform the thicknesses to the cell faces (Arakawa-C) or edges (Arakawa-B) for all models. Unfortunately, these data are not available for all CMIP6 models, and we hope the situation will improve in future CMIPs.

Surface energy fluxes in CMIP6 are generally strongly underestimated compared to the observationally constrained reference estimates, with the largest biases occurring in autumn, winter and spring. Radiative fluxes at TOA are closer to observations, but some models still show biases, especially in summer. Errors in F$_s$ and F$_{TOA}$ are closely related to the extent of the simulated sea ice area. Therefore, models with particularly large biases in their simulation of sea ice (CMCC-CM2-SR5, CAS-ESM2-0, FIO-ESM2-0, NorCPM1, FGOALS-g3) also have the largest errors in F$_s$ and F$_{TOA}$. Also problems in the models’ energy conservation may lead to errors in the net global energy budget at TOA and at the surface (Wild 2020), however those errors should be comparatively small.

As with the water budget, also for the energy budget the largest uncertainties and biases seem to be generated in the ocean. While most models are able to correctly simulate the timing of the oceanic lateral heat inflows, the inter-model spread is exceptionally large and most models show a systematic underestimation of the heat transports. Six models (BCC-CSM2-MR, BCC-ESM1, CAMS-CSM1-0, FGOALS-g3, NESM3) simulate particularly small heat transports, mostly due to temperature biases but also because of too weak simulated Barents Sea volume transports. We find strong relationships between lateral oceanic heat transports and the mean state of the Arctic. Furthermore, oceanic transports have strong effects on sea ice cover and ocean warming rates, demonstrating the importance of the mean state on projected trends.

Biases in Arctic deep waters were shown to be caused by the lack of ventilation through shelf overflows and inaccurate oceanic transports (Heuzé et al. 2023). In addition, longer spin-up times may be required as deep waters may take longer to equilibrate to the initial conditions. A more detailed assessment of oceanic transports would be necessary to determine the exact source of these biases.

Despite the use of more accurate oceanic transport estimates and the assessment of more complete budgets, it was still not possible to close the energy and water budgets for the individual models completely. Nevertheless, energy budget residuals are smaller than 2 $\hbox {Wm}^{-2}$ for most models, which is still small when compared to the inter-model spreads in most energy budget components. Small residuals could be due to both technical issues on our side and deficiencies in the models, including model coupling, physical processes and numerical schemes. More extensive evaluations of these imbalances could help to further identify and address biases and limitations, leading to improved representations of regional processes and more balanced budgets.

Furthermore, it must be reiterated that all multi-model averages were computed using all available models without any kind of model weighting, which should be applied to mitigate biases, uncertainties and discrepancies between models and provide a more balanced representation of the overall model ensemble. The results of this study can nevertheless help us to understand typical model biases in the Arctic, and using these results it may be possible to generate physically based metrics to detect outliers from the model ensemble. These metrics may prove may be useful in reducing the spread of future projections of Arctic change.

Large model spreads can be exacerbated by several sources of error. First and foremost, we used only one realisation per model, which is known to introduce a sampling error as each different realisation simulates a different possible outcome of the chaotic climate system (Wang et al. 2022). However, past studies suggested intra-model biases to be quite small compared to inter-model biases (e.g., Zanowski et al. 2021; Khosravi et al. 2022; Wang et al. 2022). We used a bootstraping approach to estimate those sampling errors and found this to be true for for most variables in our study. Also, observations similarly account for only one realisation and therefore the sampling error should be of the same value for our observational estimates. So, in most cases, biases between models and observations when looking at long-term means are very likely to be true systematic biases inherent in the model. However, for variables with larger sampling errors, like e.g. OHCT and MET, and also when looking at trends of the relatively short period of 22-years, variabilities on different time scales may introduce sampling uncertainty. In those cases, the best solution would be to look at longer time scales, whereby this oftentimes is problematic due to the length of available observations and spinup effects during the earlier part of the model simulations.

In addition, errors may be introduced by missing processes or different treatment of processes in the models. For example, as we saw in Fig. 3, the inclusion of a non-linear free surface scheme leads to biased seasonal cycles of oceanic volume transports, at least in the current generation of climate models. Errors in the calculation of energy and water budget variables have been minimised by using the native grid files of all variables where interpolation can corrupt the result.

In conclusion, the biases we find in some of the Arctics’ energy and water budgets of the evaluated models have substantial effects on the simulated mean state and changes within the system and therefore possibly also on projections of future warming of the Arctic. To obtain more realistic simulations of the Arctic and processes therein more observations would be needed to constrain the models, as well as higher resolution and improved parametrizations, as already discussed by e.g., Heuzé et al. (2023). Nevertheless, the diagnostics framework presented here can be applied to measure progress made with upcoming new versions of coupled model runs, performed, e.g., within CMIP7. The presented diagnostics may also be used to generate more process-based metrics compared to earlier studies (e.g., Brunner et al. 2020) that focused on state quantities to detect outliers from the model ensemble and therefore reduce the spread of future projections of Arctic change.

## Supplementary Information

Below is the link to the electronic supplementary material.

Supplementary file1 (PDF 336 KB)

## Data Availability

CMIP6 data is available through the Earth System Grid Federation (ESGF) website https://esgf-node.llnl.gov/search/cmip6/. ERA5 data (Hersbach et al. 2019) as well as mass-consistent energy fluxes derived from ERA5 (Mayer et al. 2021b) are available in the Copernicus Climate Change Service (C3S) Climate Data Store and DEEP-C fluxes are publicly available at https://doi.org/10.17864/1947.271.

## Appendix

See Tables 6, 7, 8.

**Table 6:** Averaged volume and heat transports through the main Arctic gateways for the 1993–2014 period

	Fram		Barents		Davis		Bering	
	Volume	Heat	Volume	Heat	Volume	Heat	Volume	Heat
ACCESS-CM2	$-$ 3.3 (0.01)	27.6 ($-$ 0.02)	2.8 ($-$ 0.09*)	54.7 ($-$ 0.04*)	$-$ 0.2 (1.26*)	3.9 (0.21*)	0.7 ($-$ 0.00)	1.1 (0.05)
ACCESS-ESM1-5	$-$ 4.2 ($-$ 0.09*)	35.6 (0.02)	3.5 ($-$ 0.02*)	85.9 ($-$ 0.01)	$-$ 0.7 (0.64*)	6.3 ($-$ 0.21*)	1.3 (0.02*)	7.0 (0.17*)
BCC-CSM2-MR	$-$ 0.7 ($-$ 0.14*)	21.8 (0.03*)	0.5 (0.21*)	5.5 (0.29*)	$-$ 0.0 (4.65)	0.3 (0.04)	$-$ 0.0 (0.04*)	0.1 ($-$ 0.00)
BCC-ESM1	$-$ 0.3 ($-$ 0.02)	20.1 (0.08*)	0.1 ($-$ 0.27*)	3.6 ($-$ 0.02)	$-$ 0.1 (1.24*)	$-$ 0.2 (0.57)	$-$ 0.0 ($-$ 0.03*)	0.1 ($-$ 0.14*)
CAMS-CSM1-0	$-$ 0.9 (0.14*)	20.7 ($-$ 0.02*)	0.8 ($-$ 0.16*)	7.9 ($-$ 0.29*)	$-$ 0.5 (0.00)	7.1 (0.07*)	0.5 ($-$ 0.08*)	$-$ 0.1 (0.04)
CanESM5	$-$ 3.7 ($-$ 0.22*)	23.4 (0.26*)	3.4 (0.18*)	63.7 (0.35*)	$-$ 0.6 (0.38*)	3.2 ($-$ 0.16*)	0.8 (0.02)	3.0 (0.52*)
CAS-ESM2-0	-	-	-	-	-	-	-	-
CESM2	$-$ 1.2 ($-$ 0.12*)	10.2 (0.12*)	1.8 (0.07*)	45.6 (0.12*)	$-$ 1.9 (0.09*)	6.2 ($-$ 0.11*)	1.1 (0.02*)	6.0 (0.38*)
CESM2-WACCM	$-$ 1.2 ($-$ 0.17*)	9.0 (0.08*)	1.6 (0.12*)	41.3 (0.10*)	$-$ 1.8 (0.07*)	6.4 ($-$ 0.01)	1.1 (0.00)	3.7 (0.44*)
CMCC-CM2-HR4	$-$ 2.5 (0.07*)	58.8 (0.15*)	3.5 ($-$ 0.01)	95.8 (0.00)	$-$ 2.7 ($-$ 0.05*)	25.9 (0.09*)	1.5 (0.01)	8.7 (0.19*)
CMCC-CM2-SR5	$-$ 3.3 (0.01)	17.1 (0.37*)	4.3 (0.01*)	106.4 (0.11*)	$-$ 2.6 ($-$ 0.05*)	8.2 ($-$ 0.03)	1.3 (0.03*)	11.9 (0.34*)
CNRM-CM6-1	$-$ 3.4 ($-$ 0.23*)	24.0 ($-$ 0.06*)	3.4 ($-$ 0.14*)	64.1 ($-$ 0.15*)	$-$ 1.6 (0.76*)	8.8 ($-$ 0.31*)	1.5 (0.01*)	7.0 (0.05)
CNRM-CM6-1-HR	$-$ 1.0 ($-$ 0.19*)	29.8 (0.30*)	1.9 (0.05*)	50.5 (0.18*)	$-$ 2.2 (0.09*)	27.2 ($-$ 0.04*)	0.9 ($-$ 0.06*)	6.6 (0.02)
EC-Earth3P-HR	$-$ 2.3 ($-$ 0.14*)	34.9 (0.25*)	2.1 (0.08*)	58.4 (0.09*)	$-$ 0.9 (0.15*)	16.2 ($-$ 0.10*)	1.1 (0.01)	6.0 ($-$ 0.08*)
EC-Earth3	$-$ 2.6 ($-$ 0.32*)	3.8 (1.80*)	3.2 (0.33*)	79.1 (0.45*)	$-$ 2.4 ($-$ 0.16*)	7.7 ($-$ 0.13*)	1.8 (0.06*)	12.6 (0.37*)
FGOALS-f3-L	$-$ 3.6 (0.11*)	6.1 (0.48*)	2.0 ($-$ 0.04*)	29.9 ($-$ 0.03*)	$-$ 0.9 (0.16*)	14.4 ($-$ 0.21*)	2.1 ($-$ 0.02*)	14.0 (0.06*)
FGOALS-g3	$-$ 1.1 (0.40*)	9.4 (0.29*)	0.6 ($-$ 0.29*)	8.2 (0.50*)	$-$ 1.4 (0.16*)	8.2 ($-$ 0.17*)	1.9 (0.01*)	6.0 (0.43*)
FIO-ESM2-0	$-$ 1.4 ($-$ 0.03*)	11.1 ($-$ 0.08*)	2.4 ($-$ 0.02)	75.5 ($-$ 0.04*)	$-$ 1.9 (0.06*)	10.5 ($-$ 0.10*)	0.9 (0.04*)	6.0 (0.43*)
GFDL-CM4	$-$ 1.7 ($-$ 0.29*)	26.1 (0.08*)	2.4 (0.14*)	69.7 (0.15*)	$-$ 1.8 (0.07*)	24.8 (0.02)	0.9 (0.02*)	1.9 (0.37*)
GFDL-ESM4	–	–	–	–	–	–	–	–
GISS-E2-1-G	–	–	–	–	–	–	–	–
HadGEM3-GC31-LL	$-$ 3.4 ($-$ 0.04*)	23.5 (0.21*)	3.8 (0.00)	83.3 (0.05*)	$-$ 1.8 (0.03*)	7.5 ($-$ 0.11*)	1.3 (0.03*)	2.4 (0.62*)
HadGEM3-GC31-MM	$-$ 2.3 ($-$ 0.17*)	41.2 (0.03)	2.5 (0.06*)	60.5 (0.09*)	$-$ 1.4 (0.07*)	17.9 (0.17*)	1.0 (0.09*)	2.9 (0.34*)
INM-CM5-0	-	-	-	-	-	-	-	-
IPSL-CM6A-LR	$-$ 4.1 ($-$ 0.13*)	2.3 (0.87*)	4.1 ($-$ 0.02*)	97.2 (0.00)	$-$ 1.3 (0.49*)	4.6 ($-$ 0.08*)	1.1 ($-$ 0.01)	2.9 (0.34*)
IPSL-CM6A-LR-INCA	$-$ 3.6 ($-$ 0.26*)	$-$ 1.1 ($-$ 1.59*)	4.0 (0.10*)	100.0 (0.08*)	$-$ 1.7 (0.24*)	3.2 (0.14*)	1.1 (0.12*)	6.6 (0.20*)
KACE-1-0-G	–	–	–	–	–	–	–	–
KIOST-ESM	–	–	–	–	–	–	–	–
MIROC-ES2L	$-$ 3.2 (0.02)	42.7 (0.10*)	2.5 ($-$ 0.07*)	71.1 (0.13*)	0.2 (0.74*)	4.7 (0.03)	0.5 ($-$ 0.03)	3.1 (0.26*)
MIROC6	$-$ 2.8 (0.02*)	40.5 (0.16*)	2.9 (0.06*)	43.7 ($-$ 0.07*)	$-$ 1.1 ($-$ 0.18*)	4.7 (0.04)	1.0 ($-$ 0.00)	3.0 (0.36*)
MPI-ESM1-2-HR	$-$ 2.8 ($-$ 0.11*)	17.5 ($-$ 0.06)	2.7 (0.18*)	70.3 (0.22*)	$-$ 0.8 ($-$ 0.10*)	8.6 (0.16*)	0.7 (0.07*)	3.3 (0.42*)
MPI-ESM1-2-LR	$-$ 2.1 ($-$ 0.32*)	26.0 ($-$ 0.22*)	2.9 (0.12*)	86.0 (0.13*)	$-$ 1.1 (0.23*)	10.7 ($-$ 0.21*)	0.6 (0.04*)	1.6 (0.52*)
MRI-ESM2-0	$-$ 2.6 ($-$ 0.25*)	9.3 ($-$ 0.29*)	*2.5 (0.15*)*	61.1 (0.22*)	$-$ 1.7 (0.00)	6.8 (0.23*)	0.8 ($-$ 0.05*)	6.6
NESM3	$-$ 0.8 ($-$ 0.29*)	10.2 ($-$ 0.11*)	1.7 (0.11*)	27.2 (0.34*)	$-$ 1.5 (0.01)	4.8 ($-$ 0.16*)	0.3 (0.05*)	$-$ 1.9 (0.21*)
NorCPM1	–	–	–	–	–	–	–	–
NorESM2-MM	–	-	–	–	–	–	–	–
SAM0-UNICON	$-$ 1.2 ($-$ 0.21*)	10.0 ($-$ 0.08*)	2.0 (0.24*)	41.0 (0.28*)	$-$ 2.0 ($-$ 0.11*)	10.0 (0.15*)	1.0 ($-$ 0.04*)	0.7 (1.37*)
TaiESM1	$-$ 1.6 ($-$ 0.12*)	8.0 ($-$ 0.30*)	2.3 (0.08*)	59.0 (0.13*)	$-$ 2.0 (0.01*)	9.8 (0.04*)	1.0 (0.04*)	2.0 (0.78*)
UKESM1-0-LL	$-$ 2.0 ($-$ 0.11*)	19.4 (0.07*)	2.8 ($-$ 0.02*)	62.7 (0.06*)	$-$ 2.2 (0.13*)	9.9 ($-$ 0.30*)	1.3 ($-$ 0.03*)	2.2 (0.59*)
**MMM**	$-$ 2.3 ($-$ 0.11)	20.2 (0.09)	2.4 (0.05)	57.4 (0.11)	$-$ 1.3 (0.11)	9.1 ($-$ 0.03)	0.8 (0.02)	3.6 (0.31)
**REF**	$-$ 2.2 ($-$ 0.10)	34.0 (0.03)	3.2 (0.07)	78.3 (0.12)	$-$ 2.4 ($-$ 0.00)	9.5 ($-$ 0.16)	1.2 ($-$ 0.02)	4.9 (0.12)
**ArcGate**	$-$ 1.4	67.6	2.2	54.7	$-$ 1.9	31.8	1.0	$-$ 2.6

Volume Transports are given in SV and heat transports in TW. REF values are calculated using the GREP reanalyses ensemble. Values in brackets show Trends in [fraction/decade]

**Table 7:** Water and energy fluxes and storage rates for the reference estimates (Ref) and the CMIP6 MMM for 1993–2014

[10$^3$ $\hbox {m}^3$/s]	Ref	CMIP6	Winkelbauer et al. (2022)	ArcGate
P-E	69.2 ± 2.5	75.9 ± 7.8	Same as Ref	–
R	127.0 ± 1.1	141.1 ± 21.4	Same as Ref	–
R$_{G}$	11.9 ± 0.4	5.6 ± 4.6	Same as Ref	–
OVT	$-$ 151 ± 43	$-$ 145 ± 139	$-$ 207	$-$ 91
OIT	$-$ 60 ± 5	$-$ 101 ± 42		$-$ 65
Res/S	$-$ 2.9	$-$ 23.4	$-$ 1.1	$-$ 52.1

$[$ $\hbox {Wm}^{-2}$ $]$	Ref	CMIP6	Mayer et al. (2019)	ArcGate
Rad$_{TOA}$	$-$ 116.7 ± 0.5	$-$ 111.6 ± 4.4	$-$ 115.8	–
F$_{S}$	$-$ 18.0 ± 3.9	$-$ 7.6 ± 2.8	$-$ 16.2	–
AET	0.1 ± 0.9	0.0 ± 0.1	$-$ 0.1	–
OHCT	0.3 ± 0.4	0.5 ± 0.4	0.3	–
MET	0.3 ± 0.1	0.2 ± 0.1	0.4	–
AHT	98.0 ± 4.0	103.9 ± 5.2	99.6	–
OHT	11.3 ± 3.7	8.2 ± 3.3	15.5	13.4
IHT	1.6 ± 0.8	2.1 ± 1.2	1.4	–
Res	$-$ 5.8	2.0	0.0	$-$ 3.6

Additionally ArcGate estimates and estimates from Winkelbauer et al. (2022) and Mayer et al. (2019) are given

**Table 8:** List of Acronyms

ArcGate	Mooring-derived data of oceanic fluxes through the Arctic gateways
BSO	Barents Sea opening
C2SMOS	Sea ice product merged from Cryosat-2 and Soil Moisture and Ocean Salinity satellites
CAA	Canadian Arctic Archipelago
CERES-EBAF	Clouds and the Earth’s Radiant Energy System-Energy Balanced and Filled
CGLORS	Ocean reanalyses from the Euro-Mediterranean Center on Climate Change
CMEMS	Copernicus Marine Environment Monitoring Service
CMIP6	Coupled Model Intercomparison Project Phase 6
EGC	East Greenlandic Current
ECMWF	European Centre for Medium-Range Weather Forecasts
ERA5	ECMWF’s fifth atmospheric reanalysis
ERA-Interim	ECMWF interim reanalysis
ESGF	Earth System Grid Federation
FOAM	Ocean reanalyses from the UK Met Office
GLORYS	Ocean reanalyses from Mercator Ocean
GRACE	Gravity Recovery and Climate Experiment
GREP	Global ocean Reanalysis Ensemble Product
MMM	Multi-model mean
NC	Norwegian Coastal Current
NEMO	Nucleus for European Modelling of the Ocean
NLFS	Non-linear free surface
NRMSE	Normalized root mean square error
ORAS5	ECMWF’s Ocean Reanalysis System 5
OSR	Ocean Reanalysis
REF	Reference data
WSC	West Spitsbergen Current
AE(T)	Atmospheric energy (tendency)
AHT	Atmospheric energy/heat transport
c$_p$	Specific heat of seawater
d$_{snow}$	Snow thickness
$\nabla \cdot F_A$	Divergence of lateral atmospheric energy transports
$\nabla \cdot F_I$	Divergence of latent heat transport associated with sea ice transports
$\nabla \cdot F_O$	Divergence of ocean heat transports
$\nabla \cdot F_{vol}$	Divergence of lateral oceanic volume fluxes
$\Delta$S$_O$	Change of ocean volume
ET	Evapotranspiration
F$_s$	Surface energy flux
F$_{TOA}$	Net radiation at the top of the atmosphere (TOA)
IHCT	Sea ice sensible heat content tendency
L$_f$	Latent heat of fusion
ME(T)	Sea ice melt energy (tendency)
$\vec {n}$	Vector normal to strait
nME	Normalised mean error
OHC(T)	Ocean heat content (tendency)
OHT	Oceanic transports of heat
OIT	Oceanic transports of ice
OVT	Oceanic transports of volume
P	Precipitation
P$_{snow}$	Snowfall rate
R	Runoff
$\rho$	Density of sea water
$\rho _{snow}$	Snow density
SIE	Sea ice extent
SIE$_B$	Sea ice extent in the Barents Sea
SIT	Sea ice thickness
$\theta$	Potential temperature
$\theta _{REF}$	Reference temperature
VIWVD	Vertical integral of divergence of moisture flux
$\vec {v}_o$	Ocean velocity vector
$\vec {v}_i$	Ice velocity vector
x	Width along the oceanic strait
x$_s$, x$_e$	Start and end point of oceanic strait
z	Ocean depth

## References

[CR1] Allan RP, Liu C, Loeb NG et al (2014) Changes in global net radiative imbalance 1985–2012. Geophys Res Lett 41(15):5588–5597. 10.1002/2014GL060962 25821270 10.1002/2014GL060962 PMC4373161

[CR2] Årthun M, Eldevik T, Smedsrud LH et al (2012) Quantifying the influence of Atlantic heat on Barents sea ice variability and retreat. J Clim 25(13):4736–4743. 10.1175/JCLI-D-11-00466.1

[CR3] Årthun M, Eldevik T, Smedsrud L (2019) The role of Atlantic heat transport in future arctic winter sea ice loss. J Clim 32(11):3327–3341. 10.1175/JCLI-D-18-0750.1

[CR4] Bacon S, Aksenov Y, Fawcett S et al (2015) Arctic mass, freshwater and heat fluxes: methods and modelled seasonal variability. Phil Trans R Soc A. 10.1098/rsta.2014.0169 10.1098/rsta.2014.0169 26347537

[CR5] Bacon S, Garabato A, Aksenov Y et al (2022) Arctic ocean boundary exchanges: a review. Oceanography. 10.5670/oceanog.2022.133

[CR6] Bao Y, Song Z, Qiao F (2020) Fio-esm version 2.0: model description and evaluation. J Geophys ResOceans. 10.1029/2019JC016036

[CR7] Bethke I, Wang Y, Counillon F et al (2021) Norcpm1 and its contribution to cmip6 dcpp. Geosci Model Dev 14(11):7073–7116. 10.5194/gmd-14-7073-2021

[CR8] Bi D, Dix M, Marsland S et al (2020) Configuration and spin-up of access-cm2, the new generation Australian community climate and earth system simulator coupled model. J South Hemisphere Earth Syst Sci. 10.1071/ES19040

[CR9] Bintanja R, Selten FM (2014) Future increases in arctic precipitation linked to local evaporation and sea-ice retreat. Nature 509:479–482. 10.1038/nature13259 24805239 10.1038/nature13259

[CR10] Blackport R, Screen JA (2020) Weakened evidence for mid-latitude impacts of arctic warming. Nat Clim Chang 10(12):1065–1066. 10.1038/s41558-020-00954-y

[CR11] Bonan DB, Feldl N, Zelinka MD et al (2023) Contributions to regional precipitation change and its polar-amplified pattern under warming. Environ Res Clim 2(3):035010. 10.1088/2752-5295/ace27a

[CR12] Boucher O, Servonnat J, Albright AL et al (2020) Presentation and evaluation of the ipsl-cm6a-lr climate model. J Adv Model Earth Syst. 10.1029/2019MS002010

[CR13] Box J, Hubbard A, Bahr D et al (2022) Greenland ice sheet climate disequilibrium and committed sea-level rise. Nat Clim Change. 10.1038/s41558-022-01441-2

[CR14] Brunner L, Pendergrass AG, Lehner F et al (2020) Reduced global warming from cmip6 projections when weighting models by performance and independence. Earth Syst Dyn 11(4):995–1012. 10.5194/esd-11-995-2020

[CR15] Cai Z, You Q, Wu F et al (2021) Arctic warming revealed by multiple cmip6 models: evaluation of historical simulations and quantification of future projection uncertainties. J Clim 34:4871–4892. 10.1175/JCLI-D-20-0791.1

[CR16] Cao J, Wang B, Yang YM et al (2018) The nuist earth system model (nesm) version 3: description and preliminary evaluation. Geosci Model Dev 11(7):2975–2993. 10.5194/gmd-11-2975-2018

[CR17] Chen HM, Li J, Su JZ et al (2019) Introduction of cams-csm model and its participation in cmip6. Adv Clim Chang Res 15(5):540. 10.12006/j.issn.1673-1719.2019.186

[CR18] Cheng L, von Schuckmann K, Abraham JP et al (2022) Past and future ocean warming. Nat Rev Earth Environ 3(11):776–794. 10.1038/s43017-022-00345-1

[CR19] Coumou D, Di Capua G, Vavrus S et al (2018) The influence of arctic amplification on mid-latitude summer circulation. Nat Commun. 10.1038/s41467-018-05256-8 10.1038/s41467-018-05256-8 PMC6102303 30127423

[CR20] Danabasoglu G, Lamarque JF, Bacmeister J et al (2020) The community earth system model version 2 (cesm2). J Adv Model Earth Syst. 10.1029/2019MS001916

[CR21] Dee DP, Uppala SM, Simmons AJ et al (2011) The era-interim reanalysis: configuration and performance of the data assimilation system. Q J R Meteorol Soc 137(656):553–597. 10.1002/qj.828

[CR22] Desportes C, Garric G, Régnier C et al (2017) CMEMS-GLO-QUID-001-026, E.U. Copernicus Marine Service Information

[CR23] Docquier D, Koenigk T (2021) A review of interactions between ocean heat transport and arctic sea ice. Environ Res Lett 16(12):123002. 10.1088/1748-9326/ac30be

[CR24] Dörr J, Årthun M, Eldevik T et al (2021) Mechanisms of regional winter sea-ice variability in a warming arctic. J Clim 34:1–56. 10.1175/JCLI-D-21-0149.1

[CR25] Döscher R, Acosta M, Alessandri A et al (2022) The ec-earth3 earth system model for the coupled model intercomparison project 6. Geosci Model Dev 15(7):2973–3020. 10.5194/gmd-15-2973-2022

[CR26] Dunne JP, Horowitz LW, Adcroft AJ et al (2020) The gfdl earth system model version 4.1 (gfdl-esm 4.1): overall coupled model description and simulation characteristics. J Adv Model Earth Syst. 10.1029/2019MS002015

[CR27] Eyring V, Bony S, Meehl GA et al (2016) Overview of the coupled model intercomparison project phase 6 (cmip6) experimental design and organization. Geosci Model Dev 9(5):1937–1958. 10.5194/gmd-9-1937-2016

[CR28] Fasullo JT, Trenberth KE (2008) The annual cycle of the energy budget. Part I: global mean and land-ocean exchanges. J Clim 21(10):2297–2312. 10.1175/2007JCLI1935.1

[CR29] Fox-Kemper B, Hewitt H, Xiao C et al (2021) Ocean, cryosphere and sea level change. Cambridge University Press, Cambridge and New York, pp 1211–1362. 10.1017/9781009157896.011

[CR30] Francis JA, Vavrus SJ (2012) Evidence linking arctic amplification to extreme weather in mid-latitudes. Geophys Res Lett. 10.1029/2012GL051000

[CR31] Garric G, Parent L, Greiner E, et al (2017) Performance and quality assessment of the global ocean eddy-permitting physical reanalysis GLORYS2V4. In: EGU General Assembly Conference Abstracts, EGU General Assembly Conference Abstracts, p 18776

[CR32] Goosse H, Kay JE, Armour KC et al (2018) Quantifying climate feedbacks in polar regions. Nat Commun. 10.1038/s41467-018-04173-0 10.1038/s41467-018-04173-0 PMC5953926 29765038

[CR33] Gosling SN, Arnell NW (2011) Simulating current global river runoff with a global hydrological model: model revisions, validation, and sensitivity analysis. Hydrol Process 25(7):1129–1145. 10.1002/hyp.7727

[CR34] Haarsma R, Acosta M, Bakhshi R et al (2020) Highresmip versions of ec-earth: Ec-earth3p and ec-earth3p-hr - description, model computational performance and basic validation. Geosci Model Dev 13(8):3507–3527. 10.5194/gmd-13-3507-2020

[CR35] Haine TW, Curry B, Gerdes R et al (2015) Arctic freshwater export: status, mechanisms, and prospects. Global Planet Change 125:13–35. 10.1016/j.gloplacha.2014.11.013

[CR36] Hajima T, Watanabe M, Yamamoto A et al (2020) Development of the miroc-es2l earth system model and the evaluation of biogeochemical processes and feedbacks. Geosci Model Dev 13(5):2197–2244. 10.5194/gmd-13-2197-2020

[CR37] He B, Bao Q, Wang X et al (2019) Cas fgoals-f3-l model datasets for cmip6 historical atmospheric model intercomparison project simulation. Adv Atmos Sci 36(8):771–778. 10.1007/s00376-019-9027-8

[CR38] Held IM, Guo H, Adcroft A et al (2019) Structure and performance of gfdl’s cm4.0 climate model. J Adv Model Earth Syst 11(11):3691–3727. 10.1029/2019MS001829

[CR39] Hersbach H, Bell B, Berrisford P et al (2020) The era5 global reanalysis. Q J R Meteorol Soc 146(730):1999–2049. 10.1002/qj.3803

[CR40] Hersbach H, Bell B, Berrisford P, et al (2019) Era5 monthly averaged data on single levels from 1979 to present. 10.24381/cds.f17050d7

[CR41] Heuzé C, Zanowski H, Karam S et al (2023) The deep arctic ocean and fram strait in cmip6 models. J Clim 36(8):2551–2584. 10.1175/JCLI-D-22-0194.1

[CR42] Hou Y, Guo H, Yang Y et al (2023) Global evaluation of runoff simulation from climate, hydrological and land surface models. Water Resour Res 59(1):e2021WR031817. 10.1029/2021WR031817

[CR43] Irving D, Hobbs W, Church J et al (2021) A mass and energy conservation analysis of drift in the cmip6 ensemble. J Clim 34(8):3157–3170. 10.1175/JCLI-D-20-0281.1

[CR44] Kelley M, Schmidt GA, Nazarenko LS et al (2020) Giss-e2.1: configurations and climatology. J Adv Model Earth Syst. 10.1029/2019MS002025 10.1029/2019MS002025 PMC7507764 32999704

[CR45] Khosravi N, Wang Q, Koldunov N et al (2022) The arctic ocean in cmip6 models: biases and projected changes in temperature and salinity. Earth’s Future 10(2):e2021EF002282. 10.1029/2021EF002282

[CR46] Knutti R (2008) Should we believe model predictions of future climate change? Philos Trans Royal Soc A Math Phys Eng Sci 366(1885):4647–4664. 10.1098/rsta.2008.0169 10.1098/rsta.2008.0169 18818153

[CR47] Kouki K, Räisänen P, Luojus K et al (2022) Evaluation of northern hemisphere snow water equivalent in cmip6 models during 1982–2014. Cryosphere 16(3):1007–1030. 10.5194/tc-16-1007-2022

[CR48] Kwok R (2018) Arctic sea ice thickness, volume, and multiyear ice coverage: losses and coupled variability (1958–2018). Environ Res Lett 13(10):105005. 10.1088/1748-9326/aae3ec

[CR49] Lauritzen P, Kevlahan N, Toniazzo T et al (2022) Reconciling and improving formulations for thermodynamics and conservation principles in earth system models (esms). J Adv Model Earth Syst. 10.1029/2022MS003117

[CR50] Lee J, Kim J, Sun MA et al (2020) Evaluation of the Korea meteorological administration advanced community earth-system model (k-ace). Asia Pac J Atmos Sci. 10.1007/s13143-019-00144-7

[CR51] Li L, Yu Y, Tang Y et al (2020) The flexible global ocean-atmosphere-land system model grid-point version 3 (fgoals-g3): description and evaluation. J Adv Model Earth Syst 12(9):e2019MS002012. 10.1029/2019MS002012

[CR52] Liu C, Allan RP, Mayer M et al (2020) Variability in the global energy budget and transports 1985–2017. Clim Dyn 55:3381–3396. 10.1007/s00382-020-05451-8

[CR53] Loeb NG, Doelling DR, Wang H et al (2018) Clouds and the earth’s radiant energy system (ceres) energy balanced and filled (ebaf) top-of-atmosphere (toa) edition-4.0 data product. J Clim 31(2):895–918. 10.1175/JCLI-D-17-0208.1

[CR54] MacLachlan C, Arribas A, Peterson KA et al (2015) Global seasonal forecast system version 5 (glosea5): a high-resolution seasonal forecast system. Q J R Meteorol Soc 141(689):1072–1084. 10.1002/qj.2396

[CR55] Madec G (2016) Nemo ocean engine—version 3.6. Note du Pole de modélisation, Institut Pierre-Simon Laplace (IPSL) 27

[CR56] Mauritsen T, Bader J, Becker T et al (2019) Developments in the mpi-m earth system model version 1.2 (mpi-esm1.2) and its response to increasing co2. J Adv Model Earth Syst 11(4):998–1038. 10.1029/2018MS001400 32742553 10.1029/2018MS001400 PMC7386935

[CR57] Mayer M, Haimberger L, Pietschnig M et al (2016) Facets of arctic energy accumulation based on observations and reanalyses 2000–2015. Geophys Res Lett 43(19):10,420-10,429. 10.1002/2016GL070557 10.1002/2016GL070557 PMC5102146 27867237

[CR58] Mayer M, Haimberger L, Edwards JM et al (2017) Toward consistent diagnostics of the coupled atmosphere and ocean energy budgets. J Clim 30(22):9225–9246. 10.1175/JCLI-D-17-0137.1

[CR59] Mayer M, Tietsche S, Haimberger L et al (2019) An improved estimate of the coupled arctic energy budget. J Clim 32(22):7915–7934. 10.1175/JCLI-D-19-0233.1

[CR60] Mayer J, Mayer M, Haimberger L (2021) Consistency and homogeneity of atmospheric energy, moisture, and mass budgets in era5. J Clim 34(10):3955–3974. 10.1175/JCLI-D-20-0676.1

[CR61] Mayer M, Vidar SL, Kjell AM et al (2021) Ocean heat content in the highnorth. J Oper Oceanogr 14(Suppl. 1):S17–S23. 10.1080/1755876X.2021.1946240

[CR62] Mayer M, Tsubouchi T, von Schuckmann K et al (2022) Atmospheric and oceanic contributions to observed nordic seas and arctic ocean heat content variations 1993–2020. J Oper Oceanogr 15(Suppl. 1):S20–S28. 10.1080/1755876X.2022.2095169

[CR63] Mayer J, Mayer M, Haimberger L (2021b) Mass-consistent atmospheric energy and moisture budget monthly data from 1979 to present derived from era5 reanalysis. 10.24381/cds.c2451f6b

[CR64] McPhee MG, Proshutinsky A, Morison JH et al (2009) Rapid change in freshwater content of the arctic ocean. Geophys Res Lett. 10.1029/2009GL037525

[CR65] Moon T, Ahlstrøm A, Goelzer H et al (2018) Rising oceans guaranteed: arctic land ice loss and sea level rise. Curr Clim Change Rep. 10.1007/s40641-018-0107-0 10.1007/s40641-018-0107-0 PMC6428231 30956936

[CR66] Mouginot J, Rignot E, Bjørk A et al (2019) Forty-six years of Greenland ice sheet mass balance from 1972 to 2018. Earth Atmos Planet Sci 116(19):9239–9244. 10.1073/pnas.1904242116 10.1073/pnas.1904242116 PMC6511040 31010924

[CR67] Muilwijk M, Smedsrud LH, Ilicak M et al (2018) Atlantic water heat transport variability in the 20th century arctic ocean from a global ocean model and observations. J Geophys Res Oceans 123(11):8159–8179. 10.1029/2018JC014327

[CR68] Onarheim IH, Årthun M (2017) Toward an ice-free barents sea. Geophys Res Lett 44(16):8387–8395. 10.1002/2017GL074304

[CR69] Pak G, Noh Y, Lee MI et al (2021) Korea institute of ocean science and technology earth system model and its simulation characteristics. Ocean Sci J. 10.1007/s12601-021-00001-7

[CR70] Park S, Shin J, Kim S et al (2019) Global climate simulated by the Seoul national university atmosphere model version 0 with a unified convection scheme (sam0-unicon). J Clim 32(10):2917–2949. 10.1175/JCLI-D-18-0796.1

[CR71] Pietschnig M, Mayer M, Tsubouchi T et al (2017) Volume and temperature transports through the main arctic gateways: a comparative study between an ocean reanalysis and mooring-derived data. Ocean Sci Discussions 2017:1–32. 10.5194/os-2017-98

[CR72] Pithan F, Jung T (2021) Arctic amplification of precipitation changes-the energy hypothesis. Geophys Res Lett 48(21):e2021GL094977. 10.1029/2021GL094977

[CR73] Proshutinsky A, Krishfield R, Timmermans ML et al (2009) Beaufort gyre freshwater reservoir: state and variability from observations. J Geophys Res Oceans. 10.1029/2008JC005104 10.1029/2019JC015281 PMC7003849 32055432

[CR74] Rabe B, Karcher M, Schauer U et al (2011) An assessment of arctic ocean freshwater content changes from the 1990s to the 2006–2008 period. Deep Sea Res Part I Oceanogr Res Papers 58(2):173–185. 10.1016/j.dsr.2010.12.002

[CR75] Rantanen M, Karpechko AY, Lipponen A et al (2022) The arctic has warmed nearly four times faster than the globe since 1979. Commun Earth Environ. 10.1038/s43247-022-00498-3

[CR76] Ricker R, Hendricks S, Kaleschke L et al (2017) A weekly arctic sea-ice thickness data record from merged cryosat-2 and smos satellite data. Cryosphere 11(4):1607–1623. 10.5194/tc-11-1607-2017

[CR77] Roullet G, Madec G (2000) Salt conservation, free surface, and varying levels: a new formulation for ocean general circulation models. J Geophys Res Oceans 105(C10):23927–23942. 10.1029/2000JC900089

[CR78] Rowland JC, Jones CE, Altmann G et al (2010) Arctic landscapes in transition: responses to thawing permafrost. EOS Trans Am Geophys Union 91(26):229–230. 10.1029/2010EO260001

[CR79] Schauer U, Beszczynska-Möller A (2009) Problems with estimation and interpretation of oceanic heat transport—conceptual remarks for the case of fram strait in the arctic ocean. Ocean Sci 5(4):487–494. 10.5194/os-5-487-2009

[CR80] Screen JA, Simmonds I (2013) Exploring links between arctic amplification and mid-latitude weather. Geophys Res Lett 40(5):959–964. 10.1002/grl.50174

[CR81] Seland Ø, Bentsen M, Olivié D et al (2020) Overview of the Norwegian earth system model (noresm2) and key climate response of cmip6 deck, historical, and scenario simulations. Geosci Model Dev 13(12):6165–6200. 10.5194/gmd-13-6165-2020

[CR82] Sellar AA, Jones CG, Mulcahy JP et al (2019) Ukesm1: description and evaluation of the u.k. earth system model. J Adv Model Earth Syst 11(12):4513–4558. 10.1029/2019MS001739

[CR83] Serreze MC, Barrett AP, Stroeve JC et al (2009) The emergence of surface-based arctic amplification. Cryosphere 3:11–19

[CR84] Shu Q, Wang Q, Song Z et al (2020) Assessment of sea ice extent in cmip6 with comparison to observations and cmip5. Geophys Res Lett 47(9):e2020GL087965. 10.1029/2020GL087965

[CR85] Shu Q, Wang Q, Årthun M et al (2022) Arctic ocean amplification in a warming climate in cmip6 models. Sci Adv 8(30):eabn9755. 10.1126/sciadv.abn9755 35895818 10.1126/sciadv.abn9755 PMC9328679

[CR86] Storto A, Masina S (2016) C-glorsv5: an improved multipurpose global ocean eddy-permitting physical reanalysis. Earth Syst m Sci Data 8(2):679–696. 10.5194/essd-8-679-2016

[CR87] Storto A, Alvera-Azcárate A, Balmaseda MA et al (2019) Ocean reanalyses: recent advances and unsolved challenges. Front Marine Sci. 10.3389/fmars.2019.00418

[CR88] Storto A, Masina S, Simoncelli S et al (2019) The added value of the multi-system spread information for ocean heat content and steric sea level investigations in the cmems grep ensemble reanalysis product. Clim Dyn. 10.1007/s00382-018-4585-5

[CR89] Stroeve J, Notz D (2018) Changing state of arctic sea ice across all seasons. Environ Res Lett 13(10):103001. 10.1088/1748-9326/aade56

[CR90] Swart NC, Cole JNS, Kharin VV et al (2019) The Canadian earth system model version 5 (canesm5.0.3). Geosci Model Dev 12(11):4823–4873. 10.5194/gmd-12-4823-2019

[CR91] Tatebe H, Ogura T, Nitta T et al (2019) Description and basic evaluation of simulated mean state, internal variability, and climate sensitivity in miroc6. Geosci Model Dev 12(7):2727–2765. 10.5194/gmd-12-2727-2019

[CR92] Trenberth KE, Fasullo JT, Mackaro J (2011) Atmospheric moisture transports from ocean to land and global energy flows in reanalyses. J Clim 24(18):4907–4924. 10.1175/2011JCLI4171.1

[CR93] Tsubouchi T, Bacon S, Naveira Garabato AC et al (2012) The arctic ocean in summer: a quasi-synoptic inverse estimate of boundary fluxes and water mass transformation. J Geophys Res Oceans. 10.1029/2011JC007174

[CR94] Tsubouchi T, Bacon S, Aksenov Y et al (2018) The arctic ocean seasonal cycles of heat and freshwater fluxes: observation-based inverse estimates. J Phys Oceanogr 48(9):2029–2055. 10.1175/JPO-D-17-0239.1

[CR95] Voldoire A, Saint-Martin D, Sénési S et al (2019) Evaluation of cmip6 deck experiments with cnrm-cm6-1. J Adv Model Earth Syst 11(7):2177–2213. 10.1029/2019MS001683

[CR96] von Schuckmann K, Cheng L, Palmer MD et al (2020) Heat stored in the earth system: where does the energy go? Earth Syst Sci Data 12(3):2013–2041. 10.5194/essd-12-2013-2020

[CR97] Walsh JE (2014) Intensified warming of the arctic: causes and impacts on middle latitudes. Global Planet Change 117:52–63. 10.1016/j.gloplacha.2014.03.003

[CR98] Wang C, Graham RM, Wang K et al (2019) Comparison of era5 and era-interim near-surface air temperature, snowfall and precipitation over arctic sea ice: effects on sea ice thermodynamics and evolution. Cryosphere 13(6):1661–1679. 10.5194/tc-13-1661-2019

[CR99] Wang YC, Hsu HH, Chen CA et al (2021) Performance of the Taiwan earth system model in simulating climate variability compared with observations and cmip6 model simulations. J Adv Model Earth Syst 13(7):e2020MS002353. 10.1029/2020MS002353

[CR100] Wang S, Wang Q, Wang M et al (2022) Arctic ocean freshwater in cmip6 coupled models. Earth’s Future 10(9):e2022EF002878. 10.1029/2022EF002878

[CR101] Wild M (2020) The global energy balance as represented in cmip6 climate models. Clim Dyn. 10.1007/s00382-020-05282-7 10.1007/s00382-020-05282-7 PMC7366598 32704207

[CR102] Williams KD, Copsey D, Blockley EW et al (2018) The met office global coupled model 3.0 and 3.1 (gc3.0 and gc3.1) configurations. J Adv Model Earth Syst 10(2):357–380. 10.1002/2017MS001115

[CR103] Winkelbauer S (2023) Straitflux. 10.5281/zenodo.10053555

[CR104] Winkelbauer S, Mayer M, Seitner V et al (2022) Diagnostic evaluation of river discharge into the arctic ocean and its impact on oceanic volume transports. Hydrol Earth Syst Sci 26(2):279–304. 10.5194/hess-26-279-2022

[CR105] Winkelbauer S, Mayer M, Haimberger L (2023) Straitflux—precise water strait fluxes on various modelling grids. [Manuscript in preparation]

[CR106] Wu T, Lu Y, Fang Y et al (2019) The Beijing climate center climate system model (bcc-csm): the main progress from cmip5 to cmip6. Geosci Model Dev 12(4):1573–1600. 10.5194/gmd-12-1573-2019

[CR107] Wu RJ, Lo MH, Scanlon BR (2021) The annual cycle of terrestrial water storage anomalies in cmip6 models evaluated against grace data. J Clim 34(20):8205–8217. 10.1175/JCLI-D-21-0021.1

[CR108] Yukimoto S, Kawai H, Koshiro T et al (2019) The meteorological research institute earth system model version 2.0, mri-esm2.0: description and basic evaluation of the physical component. J Meteorol Soc Jpn Ser II 97(5):931–965. 10.2151/jmsj.2019-051

[CR109] Zanowski H, Jahn A, Holland MM (2021) Arctic ocean freshwater in cmip6 ensembles: declining sea ice, increasing ocean storage and export. J Geophys Res Oceans 126(4):e2020JC016930. 10.1029/2020JC016930

[CR110] Zhang H, Zhang M, Jin J et al (2020) Description and climate simulation performance of cas-esm version 2. J Adv Model Earth Syst 12(12):e2020MS002210. 10.1029/2020MS002210

[CR111] Ziehn T, Chamberlain MA, Law RM et al (2020) The Australian earth system model: Access-esm1.5. J South Hemisphere Earth Syst Sci. 10.1071/ES19035

[CR112] Zuo H, Balmaseda M, Mogensen K (2015) The new eddy-permitting orap5 ocean reanalysis: description, evaluation and uncertainties in climate signals. Clim Dyn 49(3):791–811. 10.1007/s00382-015-2675-1

