# Supplementary material for: Validation of key Arctic energy and water budget components in CMIP6
Source: Clim Dyn. 2024 Feb 3;62(5):3891–926. doi: 10.1007/s00382-024-07105-5 (PMC11554900; doi:10.1007/s00382-024-07105-5)
Supplement: Supplementary file 1 — Supplementary file1 (PDF 336 KB) [file 382_2024_7105_MOESM1_ESM.pdf]

Validation of key Arctic energy and water budget components in CMIP6

Supporting material

Susanna Winkelbauer[1,2,*], Michael Mayer[1,2,3], Leopold Haimberger[1]
*susanna.winkelbauer@univie.ac.at
[1]Department of Meteorology and Geophysics, university of Vienna, Vienna, Austria
[2]b.geos, Korneuburg,Austria
[3]European Centre for Medium-Range Weather Forecasts, Bonn, Germany

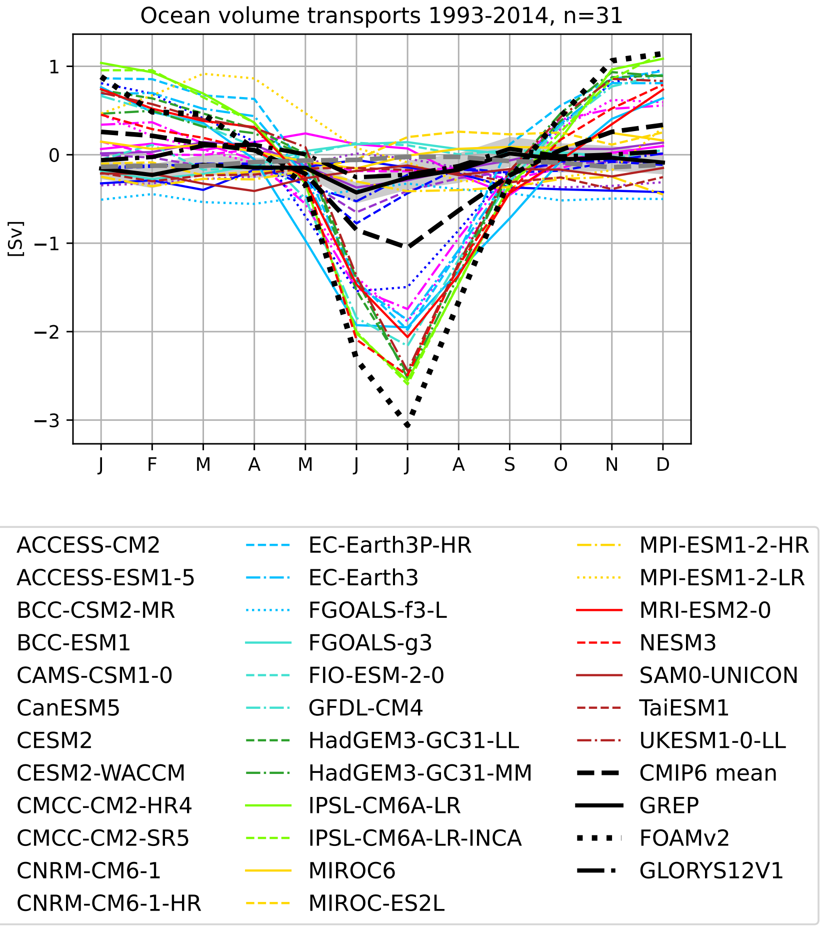

Fig. 1 Net oceanic volume transports without sea ice volume corrections for the NLFS models. Shading indicates the spread (2σ) of the GREP ensemble

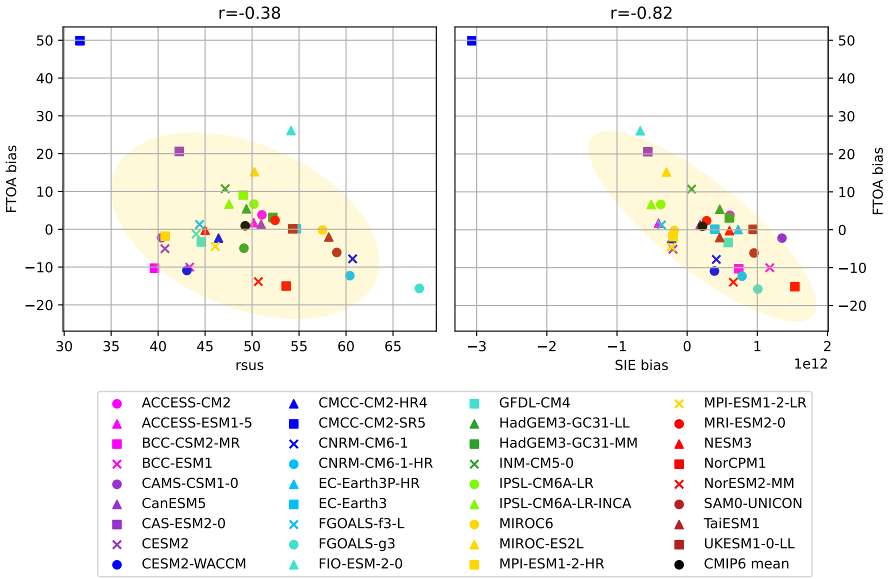

Fig. 2 Scatter diagrams of long-term averaged (1993-2014) summer FTOA bias with the reflected shortwave radiation (rsus, left panel) and the sea ice extent bias. Yellow ellipses show the 2-sigma confidence ellipses for the CMIP6 models.

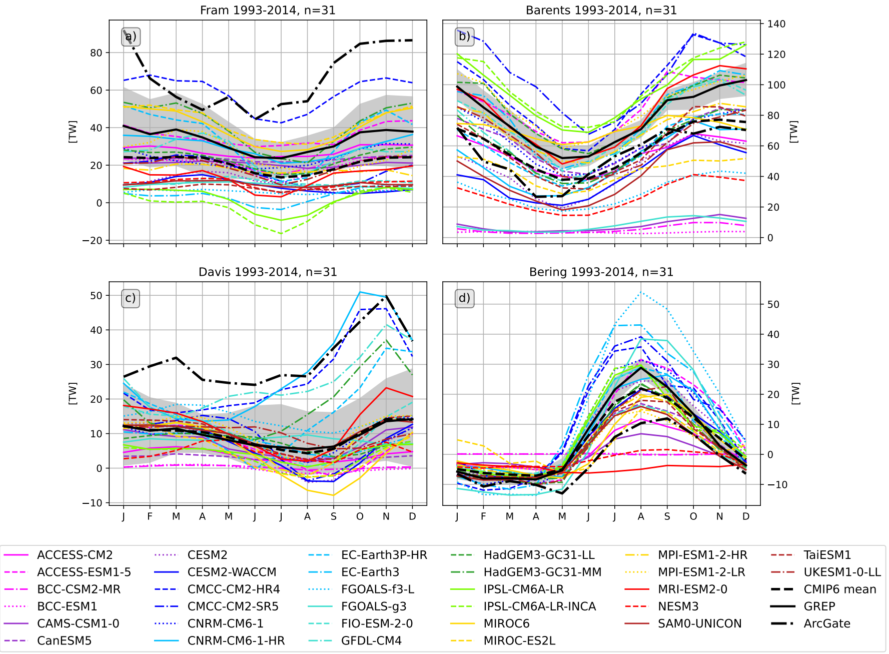

Fig. 3 Averaged annual cycles of oceanic heat transports through a) Fram Strait, b) Barents Sea Opening, c) Davis Strait and d) Bering Strait for various CMIP6 models (1993-2014), the GREP reanalyses mean (1993-2014) and ArcGate observations (2005-2010). Positive values denote transports into the Arctic. Shading indicates the spread (2σ) of the GREP ensemble

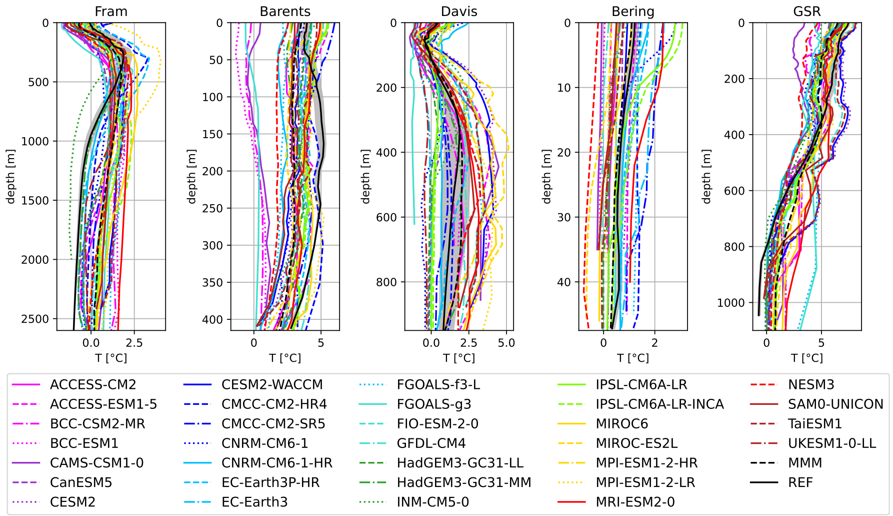

Fig. 4 Temperature profiles averaged along the main Arctic gateways. Reference values (REF) are taken from the GREP reanalyses ensemble. Shading indicates the spread (2σ) of the GREP ensemble

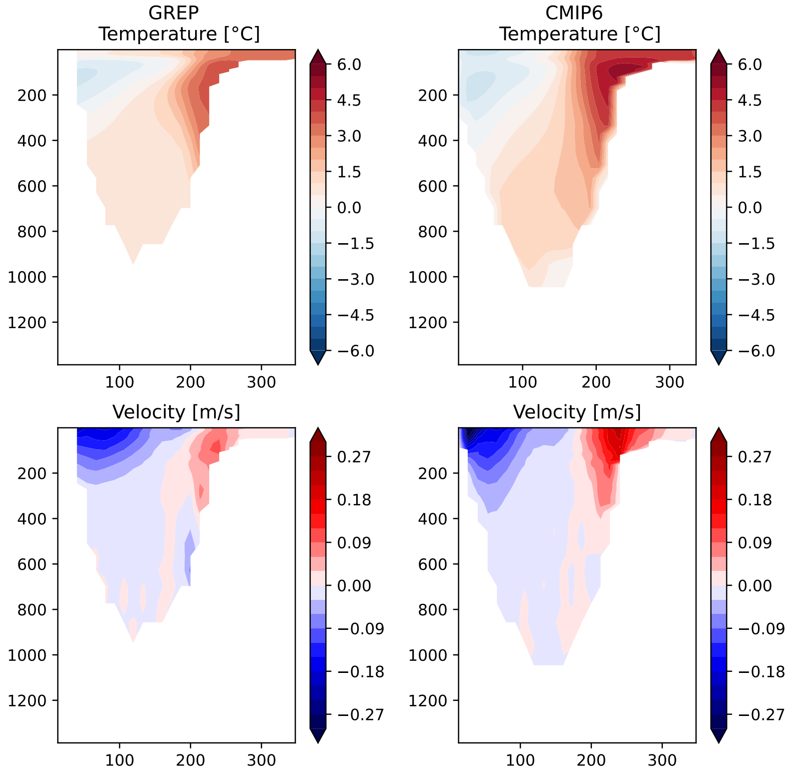

Fig. 5 Crosssections of temperatures (top) and velocities (bottom) across Davis Strait for the GREP ensemble mean (left) and the mean of all high resolution CMIP6 models.

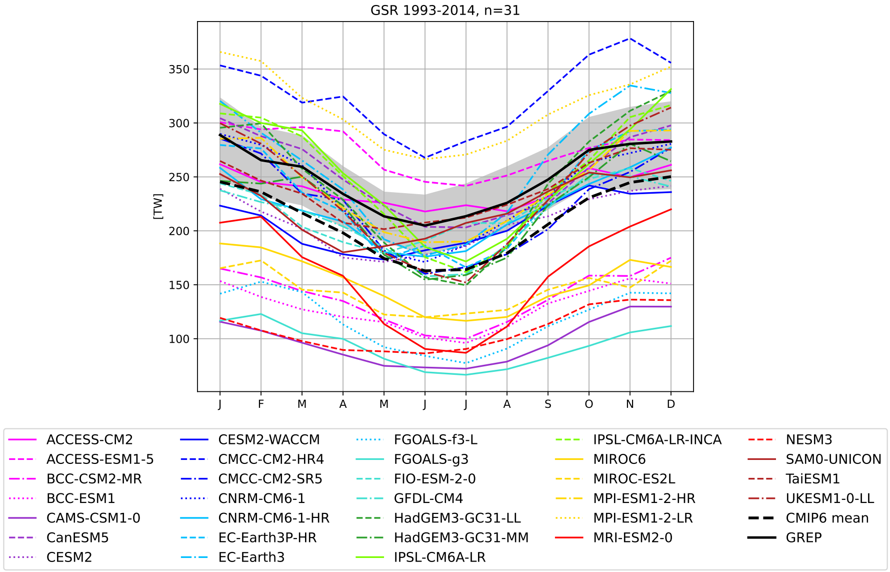

Fig. 6 Averaged annual cycles of oceanic heat transports through the Greenland Scotland Ridge (GSR) for various CMIP6 models (1993-2014), the GREP reanalyses mean (1993-2014) and ArcGate observations (2005-2010). Shading indicates the spread (2σ) of the GREP ensemble

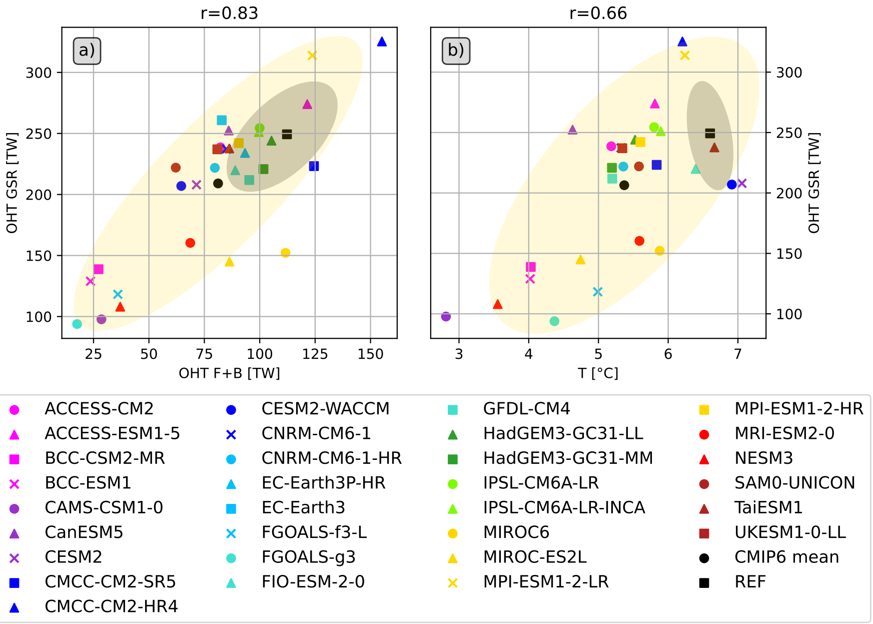

Fig. 7 Scatter diagrams of long-term (1993-2014) annual averaged a) OHT through Fram Strait + the BSO and the GSR and b) temperatures averaged across the GSR and OHT through the GSR. Reference values (REF) are calculated using the GREP ensemble. Yellow ellipses show the 2-sigma confidence ellipses for the CMIP6 models and grey ellipses for the GREP reanalyses.
